# Slow Relaxation of the Magnetization in Dysprosium–Aluminum Metallacrowns

**DOI:** 10.1021/acsomega.5c07724

**Published:** 2025-11-14

**Authors:** Simone Chicco, Elena Garlatti, Francesco Cugini, Rachel E. Rheam, Jordan R. Travis, Alyssa M. Hess, Matthias Zeller, Massimo Solzi, Stefano Carretta, Curtis M. Zaleski

**Affiliations:** † Department of Mathematical, Physical and Computer Sciences, 9370 University of Parma, Parco Area delle Scienze 7/A, 43124 Parma, Italy; ‡ INFN-Sezione di Milano Bicocca, Gruppo Collegato di Parma, 43124 Parma, Italy; § Consorzio Interuniversitario Nazionale per la Scienza e Tecnologia dei Materiali (INSTM), I-50121 Firenze, Italy; ∥ Department of Chemistry and Biochemistry, 7324 Shippensburg University, Shippensburg, Pennsylvania 17257, United States; ⊥ Department of Chemistry, 8522 Purdue University, West Lafayette, Indiana 47907, United States

## Abstract

A series of Dy^III^–Al^III^ metallacrowns (MC) with the ligand salicylhydroxamic acid (H_3_shi) were investigated for magneto-structural interactions. The four MCs demonstrate that slight variations in reaction conditions and molecular components can lead to different classes of MCs. The Dy^III^–Al^III^ MCs include an archetype **Dy** ^**III**^ **Al** ^**III**^_**4**_ **[12-MC-4]**, the dimer **Dy** ^**III**^_**2**_ **Al** ^**III**^_**8**_ **[12-MC-4]**_**2,**_ a [3.3.1] metallacryptand (MCr) **Dy** ^**III**^ **Al** ^**III**^_**6**_ **[3.3.1]**, and **Dy** ^**III**^_**2**_ **Al** ^**III**^_**6**_ **[18-MC-6]**, representing a new type of MC. **Dy** ^**III**^_**2**_ **Al** ^**III**^_**6**_ **[18-MC-6]** consists of a MC ring with six ring Al^III^ ions that have an octahedral propeller configuration with a stereoisomer pattern ΔΛΛΛΔΔ about the MC ring. The 18-MC-6 captures a [Dy^III^_2_(μ_3_-OH)_2_]^4+^ core, where each Dy^III^ ion is nine-coordinate with muffin geometry (*C*_s_). Both the static and dynamic magnetic properties of the complexes were investigated. Models of the static magnetic data reveal that **Dy** ^**III**^ **Al** ^**III**^_**4**_ **[12-MC-4]** and **Dy** ^**III**^_**2**_ **Al** ^**III**^_**8**_ **[12-MC-4]**_**2**_ do not have a significant axial ligand field, as the Dy^III^ ions have a square antiprism geometry (*D*_4d_). However, a similar analysis demonstrates that **Dy** ^**III**^ **Al** ^**III**^_**6**_ **[3.3.1]** and **Dy** ^**III**^_**2**_ **Al** ^**III**^_**6**_ **[18-MC-6]** do have a significant axial component to the ligand field, as the Dy^III^ ions in these complexes have spherical capped square antiprism (*C*_4v_) or muffin geometry, respectively. The Dy^III^ geometry differences lead to differing dynamic magnetic susceptibility behavior. **Dy** ^**III**^ **Al** ^**III**^_**4**_ **[12-MC-4]** and **Dy** ^**III**^_**2**_ **Al** ^**III**^_**8**_ **[12-MC-4]**_**2**_ do not display a frequency-dependent out-of-phase magnetic susceptibility signal in the absence of a static magnetic field. A frequency-dependent signal is observed only with the application of an 800 Oe magnetic field. However, **Dy** ^**III**^ **Al** ^**III**^_**6**_ **[3.3.1]** and **Dy** ^**III**^_**2**_ **Al** ^**III**^_**6**_ **[18-MC-6]** do exhibit SMM behavior in the absence and presence of a static magnetic field. For **Dy** ^**III**^ **Al** ^**III**^_**6**_ **[3.3.1]** and **Dy** ^**III**^_**2**_ **Al** ^**III**^_**6**_ **[18-MC-6]**, the effective energy barrier to magnetization relaxation (*U*_eff_) with an 800 Oe magnetic field is 59 ± 2 and 35 ± 2 cm^–1^ with τ_0_ = 2.5 ± 0.7 × 10^–8^ and 3.5 ± 1.1 × 10^–8^ s, respectively.

## Graphical abstract

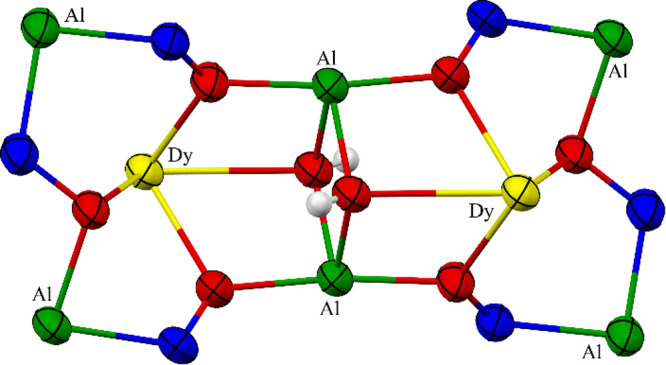

## 1 Introduction

Single-molecule magnets (SMMs) continue to attract interest since the recognition of the first SMM, a Mn_12_O_12_(acetate)_16_(H_2_O)_4_ complex, by Gatteschi, Christou, Hendrickson, and co-workers.^1–4^ Initially, research focused on 3*d*-transition metal only molecules; however, the field of SMMs soon expanded to mixed 3*d*-4*f* and 4*f*-only structures.^5–9^ While most SMMs have multiple metal centers, some only contain one paramagnetic center and are subsequently named single-ion magnets (SIMs), though the principles behind SMMs and SIMs are the same.^10,11^ SMMs have the potential to store information at cryogenic temperatures, have applications in spintronic devices, and act as qubits for quantum computing and quantum simulation.^12–18^ The properties of SMMs rely on the slow reversal of magnetization due to an effective energy barrier (*U*_eff_) to this reversal.^19^ The slow relaxation dynamics of the magnetization in these SMMs is the result of an interplay between different temperature-dependent mechanisms that arise from the molecular structure and the vibrational spectrum.^20–23^

One of the key aspects that defines SMMs is the height (i.e., size) of the energy barrier, which is directly related to the overall molecular spin state of the molecule (S_T_) and the molecular magnetoanisotropy (D). In efforts to increase the operational temperatures of SMMs, scientists have focused on increasing the S_T_ and D components through a bottom-up approach to molecular design.

Metallacrowns (MC), the metallamacrocycle analogues of organic macrocyclic crown ethers, offer a path to SMMs. MCs are a class of supramolecular species based on a ring structure with a metal–nitrogen-oxygen repeat unit.^24^ This cyclic structure generates a central cavity, and like their organic crown ether counterparts, MCs can bind various metal ions in the central cavity. Through careful choice of ring and central metal ions and surrounding ligands that form the framework of the macrocycle, MCs can be built in various sizes with 9-MC-3, 12-MC-4, and 15-MC-5 being the most common, where the first number represents the total number of atoms in the MC ring and the second number represents the number of oxygen atoms in the MC ring.^25^ Numerous MCs have been recognized as SMMs,^26–28^ including one of the first 3*d*-4*f* SMMs, a Dy^III^_6_Mn^III^_4_Mn^IV^_2_ 28-MC-10.^29^ Since that first report, lanthanide ions have been combined with an assortment of 3*d* transition metal ions to design MC-SMMs. The combination of Ln^III^ ions with manganese,^30,31^ iron,^32,33^ nickel,^34,35^ and copper^36,37^ has been a common choice and has led to SMMs from a variety of different classes of MCs, with 12-MC-4 and 15-MC-5 being the most common.^28^

One MC doctrine is the modular approach to the design of the molecules.^38^ Individual components can be substituted while retaining the overall MC structure.^39^ This can lead to the fine-tuning of the MC molecule to a particular function, such as white-light emission,^40,41^ near-infrared luminescence,^42,43^ or magnetism.^44^ For example, the first 12-MC-4 complex identified as a SMM was a Mn^II^Mn^III^_4_ system with a central Mn^II^ ion surrounded by four ring Mn^III^ ions.^45^ The overall shape of the molecule was a square due to the choice of the MC framework salicylhydroxamic acid (H_3_shi), which places the ring Mn^III^ ions 90° relative to each other.^46^ Subsequently, we showed that two of the ring Mn^III^ ions can be replaced with two Cu^II^ ions to generate a Mn^II^Mn^III^_2_Cu^II^_2_ 12-MC-4 that retains an overall square shape, and the MC maintained its SMM behavior.^47^ Moreover, we demonstrated that the central Mn^II^ ion of a 12-MC-4 with four ring Mn^III^ ions can be replaced with a Ln^III^ ion, while the overall square shape for the 12-MC-4 was maintained even though the much larger Ln^III^ ion was captured in the center of the MC cavity.^48^ We then determined that some of the Dy^III^Mn^III^_4_ 12-MC-4 complexes displayed SMM behavior, which depended on the identity of ancillary carboxylate anions.^49^ Furthermore, if the ring Mn^III^ ions were replaced with Ga^III^ or Al^III^ ions, the resulting Ln^III^Ga^III^_4_ or Ln^III^Al_4_ 12-MC-4 complexes were highly luminescent species.^50–53^ Thus, the series of 12-MC-4 complexes was systematically altered to fulfill a particular application while retaining the MC framework. In addition, we showed that planar Dy^III^Cu^II^_5_ and Ho^III^Cu^II^_5_ 15-MC-5 complexes with a central Ln^III^ ion in an eight-coordinate triangular dodecahedron (*D*_2d_) or biaugmented trigonal prism (*C*_2v_) geometry behave as weak SMMs.^54^ Recent work has focused on planar 15-MC-5 complexes based on Ln^III^ ions confined in a pentagonal bipyramidal geometry (*D*_5h_) with a strong axial component of the ligand field. By judicious choice of the axial ligands for the Ln^III^ ions, Dy^III^Cu^II^_5_, Ho^III^Ni^II^_5_, and Dy^III^Ni^II^_5_ 15-MC-5 complexes have produced the highest *U*_eff_ values for MCs thus far, with values ranging from 423 to 625 cm^–1^.^34,37,55^ These examples highlight a hallmark of MC chemistry – the modular design of molecules through careful choice of reaction conditions and of components to tailor molecules to particular properties.

Herein, we report a series of Dy^III^–Al^III^ MCs with the MC framework ligand salicylhydroxamic acid that display SMM behavior: an archetype **Dy** ^**III**^ **Al** ^**III**^_**4**_ **[12-MC-4]**, a **Dy** ^**III**^_**2**_ **Al** ^**III**^_**8**_ **[12-MC-4]**_**2**_ dimer, a **Dy** ^**III**^ **Al** ^**III**^_**6**_ **[3.3.1] MCr** (metallacryptand), and a **Dy** ^**III**^_**2**_ **Al** ^**III**^_**6**_ **[18-MC-6]**. The four molecules represent how slight variations in stoichiometric ratios between reaction components and astute ligand choices can lead to different classes of MCs. The different MC structures and coordination geometries of the central Dy^III^ ions lead to different magnetic properties; thus, providing an opportunity to investigate magneto-structural interactions. The new Dy^III^–Al^III^ systems are also compared to similar Dy^III^–Ga^III^ MCs previously investigated (see Section 3.2.2), highlighting the magneto-structural differences between the two families of compounds.

## 2 Experimental Section

### 2.1 Synthetic Materials

Dysprosium­(III) nitrate pentahydrate (99.99%) was purchased from Alfa Aesar. Salicylhydroxamic acid (>98%) was purchased from TCI America. Aluminum­(III) nitrate nonahydrate (ACS grade) was purchased from Fisher Scientific. Methanol (ACS grade) and pyridine (ACS grade) were purchased from VWR Chemicals BDH. All reagents were used as received and without further purification.

### 2.2 Syntheses

Dy^III^Na­(ben)_4_​[12-MC_Al(III)N(shi)_-4]​(H_2_O)_4_, **Dy** ^**III**^ **Al** ^**III**^_**4**_ **[12-MC-4]**, where ben^–^ is benzoate. The synthesis and X-ray crystal structure of the compound have been previously reported.^52^

{Dy^III^Na​[12-MC_Al(III)N(shi)_-4]}_2_​(iph)_4_(DMF)_2_​(H_2_O)_8_, **Dy** ^**III**^_**2**_ **Al** ^**III**^_**8**_ **[12-MC-4]**_**2**_, where iph^2–^ is isophthalate and DMF is *N*,*N*-dimethylformamide. The synthesis and X-ray crystal structure of the compound have been previously reported.^52^

[Hpy]_2_[Dy^III^​Al^III^_6_​(H_2_shi)_2_​(shi)_7_(py)_1.891_​(H_2_O)_2_], **Dy** ^**III**^ **Al** ^**III**^_**6**_ **[3.3.1] MCr** (Metallacryptand), where py is pyridine. The synthesis and X-ray crystal structure of the compound have been previously reported.^56^

Dy^III^_2_Al^III^_6_​(H_2_shi)_4_​(shi)_6_​(OH)_2_(H_2_O)_2.24_​(CH_3_OH)_1.759_​(py)_2_· 6.935py·5.631CH_3_​OH·1.2H_2_O, **Dy** ^**III**^_**2**_ **Al** ^**III**^_**6**_ **[18-MC-6]**. Dysprosium­(III) nitrate pentahydrate (0.25 mmol), aluminum­(III) nitrate nonahydrate (0.5 mmol), and H_3_shi (1 mmol) were dissolved in 30 mL of methanol, resulting in a clear, light pink solution. Then 13 mL of pyridine were added, resulting in a clear, yellow solution. The solution was stirred for 1 min and gravity filtered. No precipitate was recovered, and the filtrate remained a clear, yellow color. X-ray quality pink, block crystals were grown in 27 days by slow evaporation of the solvent. The percent yield was 2.1% based on dysprosium­(III) nitrate pentahydrate. Elemental based on loss of interstitial methanol molecules, partial loss of interstitial pyridine molecules and an additional waters of hydration: Dy_2_Al_6_​(H_2_shi)_4_(shi)_6_​(OH)_2_(H_2_O)_2.24_​(CH_3_OH)_1.759_​(py)_2_·2py·8H_2_O, C_91.759_H_97.516_​Al_6_Dy_2_​N_14_O_43.999_ [FW = 2587.32 g/mol] found % (calculated): C = 42.41 (42.60), H = 4.07 (3.80), N = 7.81 (7.58). FT-IR bands (ATR, cm^–1^): 1602, 1579, 1538, 1521, 1479, 1452, 1405, 1318, 1272, 1252, 1218, 1151, 1101, 1066, 1035, 1007, 960, 934, 865, 825, 753, 697, 680 638, 605, 564.

### 2.3 Magnetic Measurements

Magnetization and susceptibility measurements were carried out on powder samples of **Dy** ^**III**^ **Al** ^**III**^_**4**_ **[12-MC-4]**, **Dy** ^**III**^_**2**_ **Al** ^**III**^_**8**_ **[12-MC-4]**_**2**_, **Dy** ^**III**^ **Al** ^**III**^_**6**_ **[3.3.1] MCr,** and **Dy** ^**III**^_**2**_ **Al** ^**III**^_**6**_ **[18-MC-6]** using a Quantum Design MPMS-XL5 SQUID magnetometer. The powders were placed in gelatin capsules and manually compressed with the top part of each capsule inverted to minimize grain movement. Each capsule was positioned at the center of a plastic straw that was mounted onto the magnetometer sample rod. The low thermal mass of the sample holder ensures efficient heat exchange between the sample and the magnetometer sample space. Field- and temperature-dependent magnetization measurements were performed using the DC mode of the SQUID magnetometer, employing a 4 cm scan length and 3 scans per point. Magnetization as a function of magnetic field was measured from 0 to 5 T at a fixed temperature of 2 K. Temperature-dependent magnetization was recorded under a static field of 0.1 T, with a cooling sweep from 300 to 2 K at a rate of 2 K/min.

AC susceptibility measurements were conducted both in zero and under an applied static field of 0.08 T, using the AC option of the SQUID magnetometer with an AC drive field of 4 Oe. Frequency-dependent susceptibility data were collected in the 1 Hz to 1 kHz range at various temperatures between 10 and 2 K, with 0.5 K steps, using the “settle mode” and a temperature sweep rate of 0.5 K/min. The static field of 0.08 T was applied with the sample held at 2 K.

Precise temperature control and measurement in the 1.9–400 K range (with an accuracy of 0.01 K) is ensured by the magnetometer advanced Temperature Control System. This system employs two factory-calibrated negative-temperature coefficient Cernox thermometers to account for potential thermal gradients within the sample space. Below 14 K, only the field-shielded thermometer located beneath the sample tube is used in order to eliminate magnetic field interference with temperature readings.

### 2.4 X-ray Crystallography

Crystals used for single-crystal X-ray diffraction were taken from the mother liquor and were not dried. A mineral oil-coated crystal of **Dy** ^**III**^_**2**_ **Al** ^**III**^_**6**_ **[18-MC-6]** was mounted on a MicroMesh MiTeGen micromount and transferred to the diffractometer. The data were collected on a Bruker AXS D8 Quest diffractometer equipped with a solid-state CMOS area detector and a fine focus sealed tube X-ray source using Mo Kα radiation (λ = 0.71073 Å) monochromated with a Triumph curved graphite crystal. All data were collected at 150 K, and data collection and cell refinement were performed using APEX3 (version 2018.1-0) and SAINT+ embedded in APEX3, respectively.[Fn fn1] The data were scaled and corrected for absorption with SADABS as built into APEX3.^57^ Space groups were assigned using the SHELXTL suite of programs.^58^ The structures were solved using direct methods with SHELXS-97 and refined using least-squares refinements based on F^2^ with SHELXL-2018/3 and the graphical interface SHELXLE.[Fn fn2] ^,^ ^59,60^ Additional crystallographic data and experimental parameters are provided in Table S1 and the individual CIF of the compound. Important bond distances are provided in Table S2.

## 3 Results and Discussion

### A Synthesis and Characterization

The synthesis and single-crystal X-ray structures of **Dy** ^**III**^ **Al** ^**III**^_**4**_ **[12-MC-4]**,^52,53^ **Dy** ^**III**^_**2**_ **Al** ^**III**^_**8**_ **[12-MC-4]**_**2**_,^52,53^ and **Dy** ^**III**^ **Al** ^**III**^_**6**_ **[3.3.1] MCr** ^56^ (Figure 1) have been previously reported in extensive detail. Thus, only brief descriptions will be provided. **Dy** ^**III**^ **Al** ^**III**^_**4**_ **[12-MC-4]** and **Dy** ^**III**^_**2**_ **Al** ^**III**^_**8**_ **[12-MC-4]**_**2**_ are both based on a 12-MC-4 scaffold using the common MC ligand salicylhydroxamic acid (Figure 1a). For **Dy** ^**III**^ **Al**_**4**_ **[12-MC-4],** a slightly domed 12-MC-4 framework consists of 4 ring Al^III^ ions and 4 salicylhydroximate (shi^3–^) ligands that bind a central Dy^III^ ion on the convex side of the dome and a central Na^+^ ion on the concave side. The molecule possesses the typical 12-MC-4 framework with an [Al–N–O] repeat unit that recurs four times to generate a square-shaped metallamacrocycle with a central cavity lined with the oxime oxygen atoms of the shi^3–^ ligands. The central Dy^III^ ion binds to the four oxime oxygen atoms of the MC cavity, and four benzoate anions further tether the Dy^III^ ion to the MC as the carboxylate groups of the benzoate anions form bridges between each ring Al^III^ ion and the central Dy^III^ ion. A SHAPE analysis^61–63^ revealed that the eight-coordinate Dy^III^ ion has a square antiprism geometry (*D*_4d_).^64^ If the benzoate bridges are replaced by the dicarboxylate anion isophthalate, two Dy^III^Al^III^_4_ 12-MC-4 units are joined to form the dimer found in **Dy** ^**III**^_**2**_ **Al** ^**III**^_**8**_ **[12-MC-4]**_**2**_ (Figure 1b). Each central Dy^III^ ion maintains a coordination number of eight with a square antiprism geometry. The Dy^III^–Dy^III^ distance in the dimer is 7.12 Å. The Al^III^ ions of both **Dy** ^**III**^ **Al** ^**III**^_**4**_ **[12-MC-4]** and **Dy** ^**III**^_**2**_ **Al** ^**III**^_**8**_ **[12-MC-4]**_**2**_ are six-coordinate with octahedral geometry (*O*_h_).^65^ While still utilizing H_3_shi, **Dy** ^**III**^ **Al** ^**III**^_**6**_ **[3.3.1] MCr** represents a three-dimensional MC also known as a metallacryptand, MCr.^66–68^ A three-dimensional cavity is built around the nine-coordinate central Dy^III^ ion (Figure 1c). The MCr shell is composed of seven shi^3–^ ligands, which provide the metallacryptand linkages. The [3.3.1] nomenclature is analogous to that used for organic cryptands and refers to the number of oxygen atoms in each Al–N–O chain that surrounds the central Dy^III^ ion. In the case of **Dy** ^**III**^ **Al** ^**III**^_**6**_ **[3.3.1] MCr**, there are three chains with two long O–N–Al–O–N–Al–O–N linkages and one short N–O linkage. Two singly deprotonated H_2_shi^–^ ligands bind to the central Dy^III^ ion but do not contribute to the MCr linkages. As determined by a SHAPE analysis, the nine-coordinate Dy^III^ ion has a spherical capped square antiprism geometry (*C*_4v_).^69,70^ Each Al^III^ ion is six-coordinate with an octahedral geometry. Two of the Al^III^ ions have a *trans*-configuration of two different shi^3–^ ligands. The other four Al^III^ ions have a *cis*-arrangement of three different shi^3–^ ligands, which provide a propeller configuration. Thus, there are four stereocenters in the MCr with two Λ and two Δ Al^III^ stereoconfigurations.

**Figure 1:**
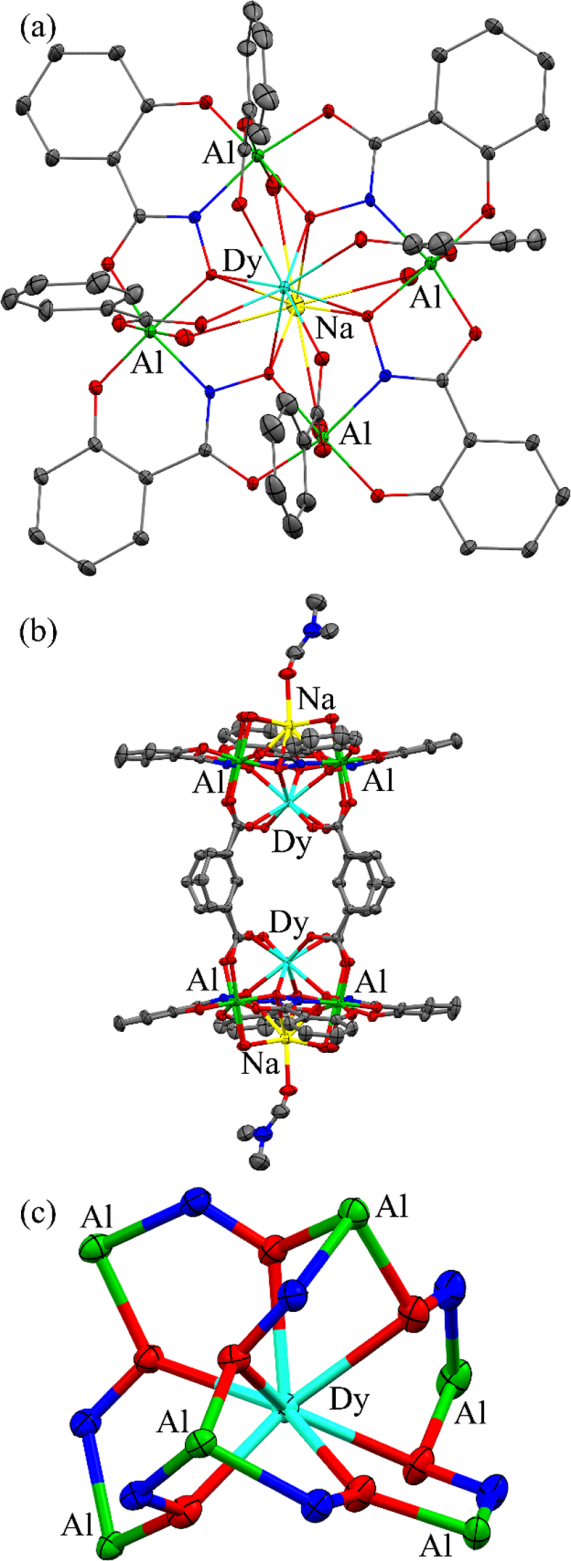
X-ray structures of (a) **Dy** ^**III**^ **Al** ^**III**^_**4**_ **[12-MC-4]**, (b) **Dy** ^**III**^_**2**_ **Al** ^**III**^_**8**_ **[12-MC-4]**_**2**_, and **(c) Dy** ^**III**^ **Al** ^**III**^_**6**_ **[3.3.1] MCr** with only the metallacryptate core shown (all other atoms were omitted for clarity). The ellipsoid plots are at the 50% level. Hydrogen atoms, disordered atoms, and solvent molecules have been omitted for clarity. Color scheme: Dy^III^—aqua, Al^III^—green, Na^+^—yellow, oxygen—red, nitrogen—blue, and carbon—gray.

The synthesis and characterization of **Dy** ^**III**^_**2**_ **Al** ^**III**^_**6**_ **[18-MC-6]** have not been previously reported. The synthesis is similar to that of the other three compounds, but some key differences lead to the generation of an 18-MC-6. For both **Dy** ^**III**^ **Al** ^**III**^_**4**_ **[12-MC-4]** and **Dy** ^**III**^_**2**_ **Al** ^**III**^_**8**_ **[12-MC-4]**_**2**_, a strict ratio of 1:4:4 between Dy­(NO_3_)_3_:Al­(NO_3_)_3_:H_3_shi with different carboxylate anions leads to the formation of a 12-MC-4 framework that maintains the 1:4:4 ratio between the central Ln^III^ ion, the ring Al^III^ ions, and the shi^3–^ ligands. For **Dy** ^**III**^ **Al** ^**III**^_**6**_ **[3.3.1] MCr**, the Dy­(NO_3_)_3_:Al­(NO_3_)_3_:H_3_shi ratio is changed to 1:6:9 with the addition of the weak bases triethylamine and pyridine. This leads to an MCr that maintains a 1:6:9 ratio among the central Dy^III^ ion, the ring Al^III^ ions, and the shi^3–^/H_2_shi^–^ ligands (7 shi^3–^ and 2 H_2_shi^–^). For **Dy** ^**III**^_**2**_ **Al** ^**III**^_**6**_ **[18-MC-6]**, the Dy­(NO_3_)_3_:Al­(NO_3_)_3_:H_3_shi ratio is altered to 1:2:4 with the addition of pyridine as the only base. This leads to an MC with a 1:3:5 ratio among the central Dy^III^ ions, the ring Al^III^ ions, and the shi^3–^/H_2_shi^–^ ligands (6 shi^3–^ and 4 H_2_shi^–^). In this case, the ratio in the resulting molecule is different than that of the starting materials. At this time, we do not have a conclusive explanation for this discrepancy.

The **Dy** ^**III**^_**2**_ **Al** ^**III**^_**6**_ **[18-MC-6]** (Figure 2) consists of two Dy^III^ and six Al^III^ ions (total 24+ charge) that are counterbalanced by four singly deprotonated salicylhydroximate anions (H_2_shi^–^), six triply deprotonated salicylhydroximate anions (shi^3–^), and two μ_3_-hydroxide anions (total 24- charge). Solvent water, pyridine, and methanol molecules complete the coordination of some of the metal ions. In addition, there are disordered interstitial water, methanol, and pyridine solvent molecules. The CIF contains a detailed explanation of the treatment of the disordered solvate molecules.

**Figure 2:**
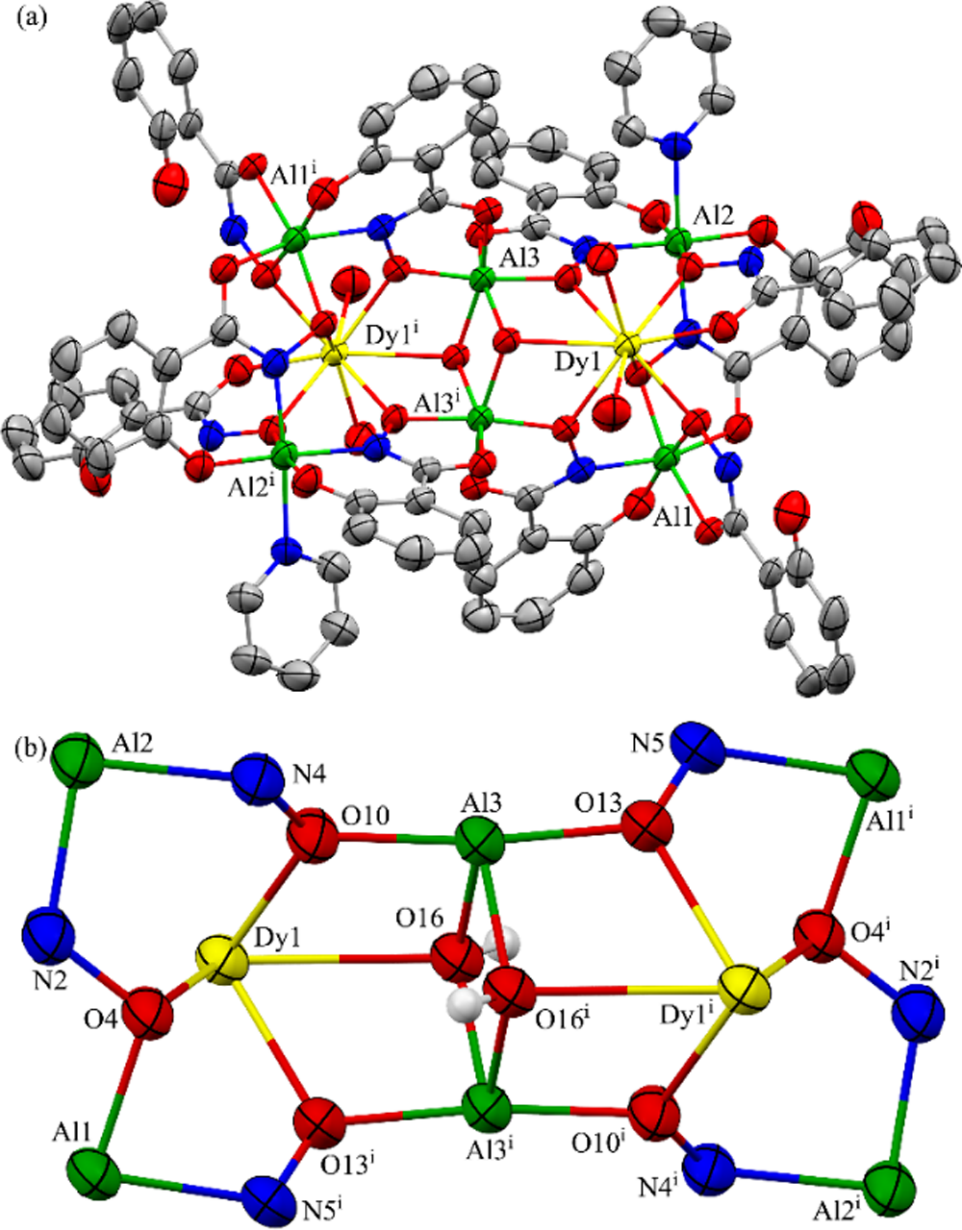
(a) X-ray structure of **Dy** ^**III**^_**2**_ **Al** ^**III**^_**6**_ **[18-MC-6]**. Hydrogen atoms, disorder, and solvent molecules have been omitted for clarity. (b) Highlight of the 18-MC-6 ring with the captured [Dy^III^_2_(μ_3_-OH)_2_]^4+^ core. Hydrogen atoms were placed on the μ_3_-OH anions. The ellipsoid plots are at the 50% level. Color scheme: Dy^III^—yellow, Al^III^—green, oxygen—red, nitrogen—blue, and carbon—gray [symmetry code: (i) −*x* + 1, −*y* + 1, −*z*].

The main structure of the **Dy** ^**III**^_**2**_ **Al** ^**III**^_**6**_ **[18-MC-6]** is positioned about an inversion center located between the μ_3_-OH anions that bridge between two Al^III^ and one Dy^III^ ions. The overall connectivity of the molecule is that of an 18-MC-6, as the Al^III^ ions form a ring about the outer perimeter of the structure, and N–O bridges exist between each Al^III^ ion (Figure 2b). However, the connectivity does not strictly match that of archetypal MCs. In standard MC systems, the N–O linkage does not reverse about the ring to O–N.^24^ In the **Dy** ^**III**^_**2**_ **Al** ^**III**^_**6**_ **[18-MC-6],** the connectivity does not strictly remain −[Al–N–O]–. Instead, the connectivity in the **Dy** ^**III**^_**2**_ **Al** ^**III**^_**6**_ **[18-MC-6]** follows a pattern of −[Al1–O–N–Al2–N–O–Al3–O–N]– that recurs twice to generate the 18-MC-6. In this pattern, the middle Al^III^ ion does not bind to an oxime atom of shi^3–^; thus, the standard MC model is not produced. In the structure, only the six shi^3–^ ligands provide nitrogen–oxygen connectivity between the Al^III^ centers of the 18-MC-6 ring. The four H_2_shi^–^ ligands form bridges between the Dy^III^ and Al^III^ ions, but the H_2_shi^–^ ligands do not participate in the MC connectivity. In essence, the H_2_shi^–^ ligand helps tether the Dy^III^ ions to the MC cavity similar to carboxylate anions such as acetate do in archetypal Ln^III^[12-MC_M(III)N(shi)_-4] structures, where M^III^ = Mn, Al, or Ga.^48,50,52^ Moreover, archetypal MCs capture a single metal ion in the central cavity.^24^ However, for the **Dy** ^**III**^_**2**_ **Al** ^**III**^_**6**_ **[18-MC-6]**, a [Dy^III^_2_(μ_3_-OH)_2_]^4+^ core comprises the center of the molecule. The capture of a bimetallic core is reminiscent of other nonarchetypal MCs based on lanthanide and manganese ions. A Dy^III^_4_Mn^III^_4_ 16-MC-6 structure with a heterobimetallic MC ring and a [Dy^III^-O–Mn^III^–N–O–Mn^III^–N–O] connectivity also captures a [Dy^III^_2_(μ_3_-OH)_2_]^4+^ core.^71^ Furthermore, a Ho^III^_4_Mn^III^_6_ 22-MC-8 structure with a heterobimetallic MC ring and a [Ho^III^-O–N–Mn^III^–O–Mn^III^–N–O–Mn^III^–N–O] connectivity captures a [Ho^III^_2_(μ_3_-OH)_2_]^4+^ core.^72^

The captured Dy^III^ ions of the [Dy^III^_2_(μ_3_-OH)_2_]^4+^ core are nine-coordinate. A SHAPE analysis (*SHAPE 2.1*; Table S3) of the geometry reveals that the lowest Continuous Shapes Measures (CShM) value is for a muffin configuration (*C*_s_; CShM = 1.385; Figure S1), though the CShM value for a spherical capped square antiprism (*C*_4v_; 1.405) is comparable. A muffin geometry is best described as a shape with a trigonal base, a pentagonal equatorial plane, and a single-point vertex.^69,70^ The Dy^III^–Dy^III^ distance across the core is 6.29 Å. Each Dy^III^ ion binds to three shi^3–^ ligands, two H_2_shi^–^ ligands, a μ_3_-OH anion, an oxygen atom of a nondisordered water molecule, and an oxygen atom of a disordered solvent molecule (water or methanol). The three shi^3–^ ligands and one of the H_2_shi- ligands donate an oxime oxygen atom in a monodentate fashion to the Dy^III^ ion. The other H_2_shi^–^ ligand binds in a bidentate fashion by using the carbonyl and oxime oxygen atoms of the ligand to form a five-membered chelate ring. The oxime oxygen atoms of the shi^3–^ and H_2_shi^–^ ligands also bridge to the ring Al^III^ ions (Al1–Al3), helping to secure the Dy^III^ ions to the MC cavity. In addition, each Dy^III^ ion binds to a μ_3_-OH anion that bridges to Al3 and the other Al3 related by the inversion center of the molecule. Lastly, the coordination sphere of each Dy^III^ ion is completed by two solvent oxygen atoms, an oxygen atom (O17) of a nondisordered water molecule, and an oxygen atom of a disordered solvent molecule, with all solvent molecules associated with O19. The disordered solvent molecule is either a water molecule or a methanol molecule with its methyl group disordered over two positions. The occupancy of the water molecule was refined to 0.120(3), while the two positions of the methyl groups of the methanol molecule were refined to 0.6223(17) and 0.257(3).

The ring Al^III^ ions are six-coordinate with an octahedral geometry (Table S4 and Figure S2). The three unique Al^III^ ions of the ring each have slightly different coordination spheres. For Al1, the coordination consists of three bidentate ligands in a propeller configuration with Δ stereoisomerism. For the aluminum ion Al1^i^, which is related by the inversion center [symmetry code: (i) −*x* + 1, −*y* + 1, −*z*], the stereoisomerism is Λ. The propeller is constructed by one bidentate H_2_shi^–^ ligand that binds with the oxime and carbonyl oxygen atoms to form a five-membered chelate ring, a shi^3–^ ligand that forms a similar five-membered chelate ring, and a shi^3–^ ligand that binds with the oxime nitrogen atom and phenolate oxygen atom to form a six-membered chelate ring. For Al2, the coordination consists of two *cis* bidentate shi^3–^ ligands, an oxime oxygen atom of a H_2_shi^–^, and a nitrogen atom of a pyridine molecule that is disordered over two positions with occupancy rates of 0.538(16)–0.462(16). Both shi^3–^ ligands form six-membered chelate rings by binding with the oxime nitrogen atom and phenolate oxygen atom of the ligands. The *cis* configuration of the ligands about Al2 imparts a Λ-like stereoisomerism to the metal center and a Δ-like stereoisomerism for the symmetry equivalent Al2^i^. For Al3, the coordination is composed of two *cis* bidentate shi^3–^ ligands and two oxygen atoms of *cis* μ_3_-OH anions. Both shi^3–^ ligands form five-membered chelate rings by binding with the oxime and carbonyl oxygen atoms of the ligands. The *cis* configuration of the ligands imparts a Λ-like stereoisomerism to the metal center and a Δ-like stereoisomerism for the symmetry equivalent Al3^i^. Thus, the stereoisomerimetric pattern of the Al^III^ ions [Al1–Al2–Al3–Al1^i^–Al2^i^–Al3^i^] about the 18-MC-6 ring is ΔΛΛΛΔΔ.

### B Magnetometry and Model Hamiltonian

The static and dynamic magnetic properties of **Dy** ^**III**^ **Al** ^**III**^_**4**_ **[12-MC-4]**, **Dy** ^**III**^_**2**_ **Al** ^**III**^_**8**_ **[12-MC-4]**_**2**_, **Dy** ^**III**^ **Al** ^**III**^_**6**_ **[3.3.1] MCr**, and **Dy** ^**III**^_**2**_ **Al** ^**III**^_**6**_ **[18-MC-6]** were investigated, and the experimental results were modeled with a spin Hamiltonian framework, as detailed below.

In the complexes investigated in this work, the central magnetic ion hosted in the MCs/MCr cavity is a Dy^III^ ([Xe] 6*s* ^2^ 4*f* ^9^) with a ground configuration ^6^ *H*_15*/*2_ for the free ion (*S* = 5*/*2, *L* = 5 and *J* = 15*/*2). In the weak field approximation (dominant spin–orbit coupling), *J* is a good quantum number, and the crystal field Hamiltonian acts on the |*SLJM*⟩ multiplet. The spin Hamiltonian of such a system is defined as follows:

$$H_{m}=H_{Z_{1,2}}+H_{{\mathrm{CF}}_{1,2}}+H_{\mathrm{dip}}=-\sum_{i=1,2}\mu_{B}g_{{\mathrm{Dy}}_{i}}S_{i}\cdot B_{i}+\sum_{i}^{N}\sum_{k=2,4,6}\sum_{q=-k}^{k}B_{k_{i}}^{q}\theta_{k}{\hat{O}}_{k_{i}}^{q}+S_{1}\cdot J_{12}\cdot S_{2}$$ 1

The first term, $H_{Z}$ , represents the Zeeman coupling, which accounts for the interaction with the applied static magnetic field that splits the ground-state Kramers degeneracy. The second term, $H_{\mathrm{CF}}$ , is the crystal field contribution, describing the effect of the ligand field environment on the energy levels of the |*SLJM*⟩ multiplets. Here, the crystal field potential is expressed using Steven’s equivalent operators *Ô*_*k*_*i*__^*q*^, derived from the ligand field symmetry of the Dy^III^ ions, with *B*_*k*_*i*__^*q*^ crystal field parameters and θ_*k*_ operator equivalent factors (tabulated for lanthanides in |*J,m*_*J*_⟩ basis^73^). The third term in eq 1 describes the interaction between the magnetic ions. Although this term can reasonably be assumed to be negligible for intermolecular interactions, it cannot be ignored for dimers, where intramolecular interactions occur over much shorter distances. In this case, through-space dipolar coupling is the most appropriate mechanism to describe the interaction, as the significant spatial separation between ions (even within the same molecule) strongly suppresses exchange-like coupling mechanisms. The dipolar coupling is characterized by an interaction coefficient *J*_12_^dip^, between the two magnetic centers, that in the point dipole approximation is written as

$$J_{12}^{\mathrm{dip}}=\frac{\mu_{B}^{2}}{R^{3}}\left[\left(g_{1}\cdot g_{2}-3\frac{\left(g_{1}\cdot R)(R\cdot g_{2}\right)}{|R{|}^{2}}\right)\right]$$ 2

where ***R*** is the vector that connects the two magnetic ions.

For each complex, magnetometry and susceptibility measurements were collected on powder samples with a MPMSXL5 SQUID magnetometer (Quantum Design). The magnetization was measured as a function of temperature, *M*(*T*), under a direct current (DC) applied magnetic field of 10 kOe from 300 to 2 K. Field-dependent magnetization measurements, *M*(*H*), were collected at 2 K by changing the applied magnetic field from 0 to 5 T (Figure 3). The temperature and field dependences of the system magnetization were simultaneously fitted for all compounds using the model spin Hamiltonian of eq 1 (Figure 3). The fitting procedure was performed using the PHI program.^74^

**Figure 3:**
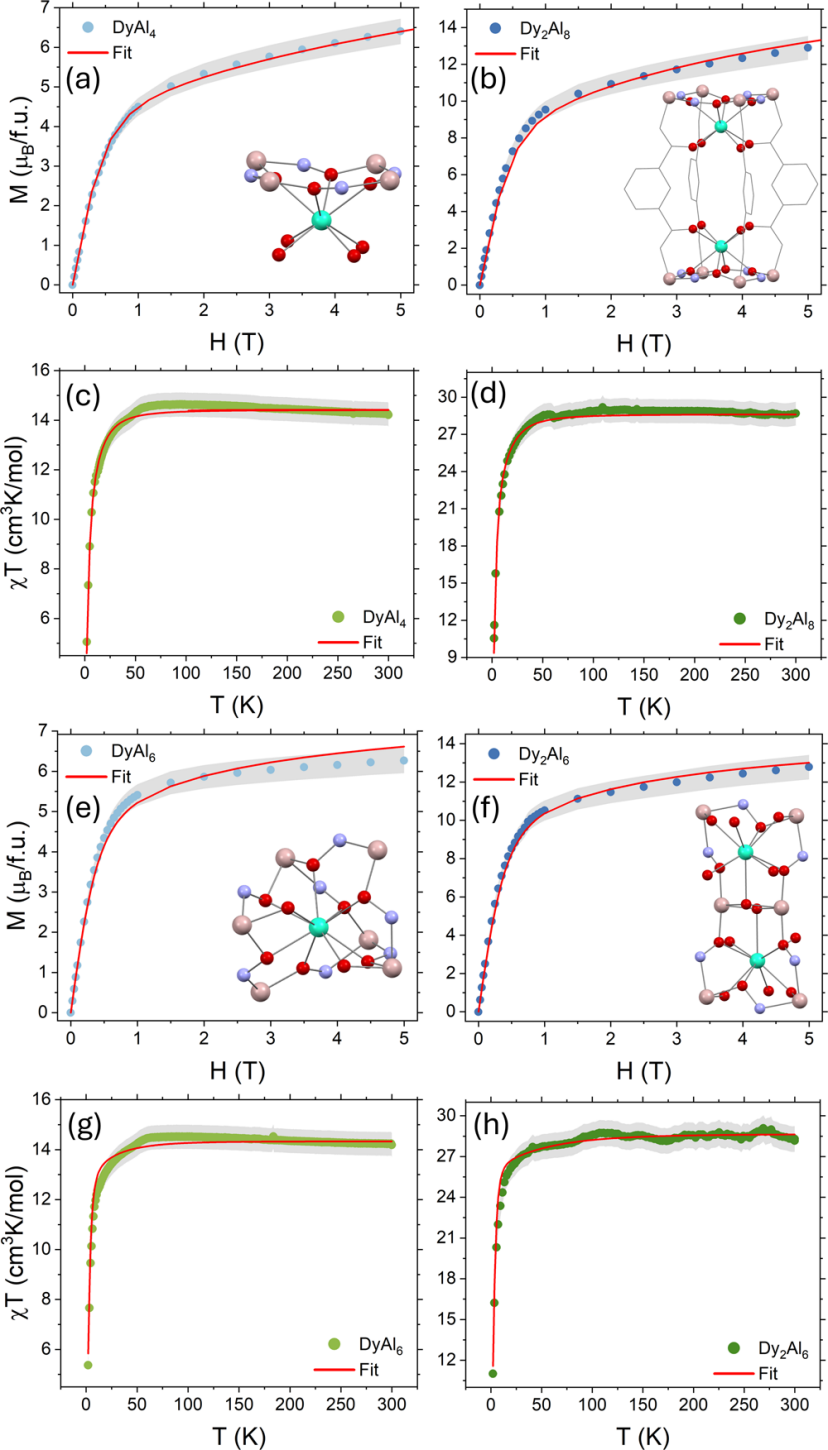
(a, b, e, f) Field dependence of the magnetization *M*(*H*), measured at 2 K, and (c, d, g, h) temperature dependence of the magnetic susceptibility product with temperature χ*T* measured in an applied static field of 10 kOe. Inset of (a, b, e, f): a sketch of the corresponding molecular structure of **Dy** ^**III**^ **Al** ^**III**^_**4**_ **[12-MC-4]**, **Dy** ^**III**^_**2**_ **Al** ^**III**^_**8**_ **[12-MC-4]**_**2**_, **Dy** ^**III**^ **Al** ^**III**^_**6**_ **[3.3.1] MCr**, and **Dy** ^**III**^_**2**_ **Al** ^**III**^_**6**_ **[18-MC-6]**. All data are corrected for the contribution of the sample diamagnetism, estimated from Pascal’s constants. The red lines represent the best-fit result obtained with Hamiltonian (1). Shaded error bars are estimated within a reasonable approximation of the cumulative experimental uncertainty to 5% of the measured values.[Fn fn3]

Finally, alternating current (AC) magnetic susceptibility measurements were conducted to assess the magnetization dynamics of these complexes (Figure 4). The AC susceptibility was measured by varying the frequency of the AC field in the 2–10 K temperature range under zero and 800 Oe static magnetic field. Comparison of the results obtained reveals the main differences in the dynamic magnetic behavior between the structures with different coordination geometries about the Dy^III^ ions.

**Figure 4:**
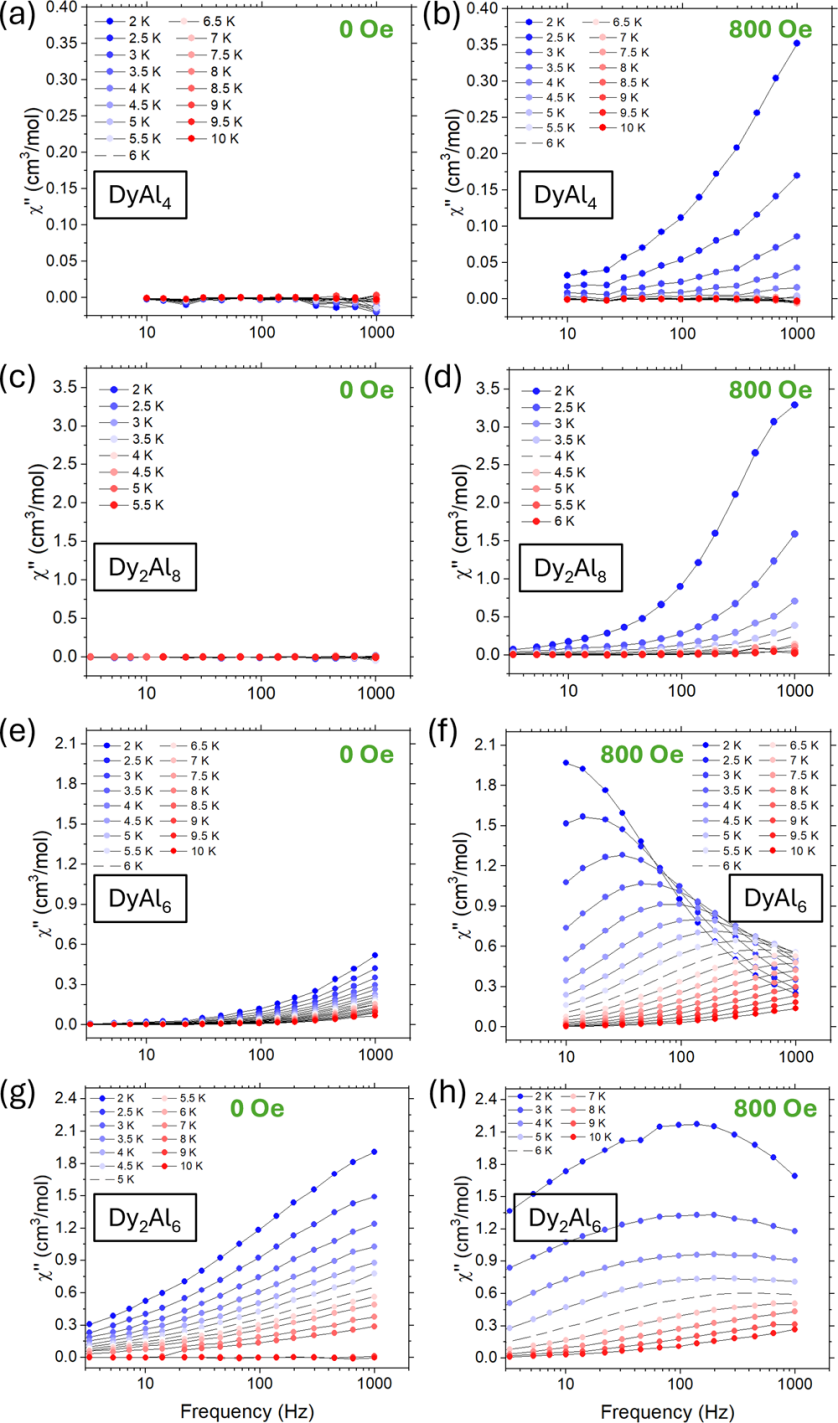
Frequency dependence of the out-of-phase susceptibilities for **Dy** ^**III**^ **Al** ^**III**^_**4**_ **[12-MC-4]**, **Dy** ^**III**^_**2**_ **Al** ^**III**^_**8**_ **[12-MC-4]**_**2**_, **Dy** ^**III**^ **Al** ^**III**^_**6**_ **[3.3.1] MCr**, and **Dy** ^**III**^_**2**_ **Al** ^**III**^_**6**_ **[18-MC-6]** (specified in inset), measured at different temperatures in zero (a, c, e, g) and an applied (800 Oe,[b, d, f, h]) static field.

#### 3.2.1 Static and Dynamic Magnetic Behavior

In the case of **Dy** ^**III**^ **Al** ^**III**^_**4**_ **[12-MC-4],** the geometry about the eight-coordinate Dy^III^ ion is square antiprism (SAP), which is indicative of a *D*_4d_ coordination polyhedron with tetragonal symmetry. For exact *D*_4d_ coordination, the Steven’s operator set necessary to describe the crystal field simplifies from the broader category of tetragonal symmetries to only include the axial terms:

$$H_{\mathrm{CF}}=B_{0}^{2}{\hat{O}}_{0}^{2}+B_{0}^{4}{\hat{O}}_{0}^{4}+B_{0}^{6}{\hat{O}}_{0}^{6}$$

­(Table S5). Further details on the molecular structure, obtained from X-ray diffraction, reveal that the characteristic cubic angle of SAP θ_c_ = 54.74°, for which the *B*_0_^2^ term vanishes, is here slightly distorted and even between the upper and lower pyramids of the SAP geometry. Therefore, in this regime in which the cubic angle approaches the condition in which *B*_0_^2^ vanishes and given its nonunivocal prolate or oblate deformation, a reasonable approximation that also reduces the number of free parameters is to model the CF assuming an effective exact SAP with only the *B*_0_^4^ and *B*_0_^6^ operators. The other characteristic angle of the SAP geometry, that is, the twist angle between the two base planes ϕ_*D*4d_ = 45° is here only slightly reduced to ϕ = 41.8° and supports the assumption of an effective ideal *D*_4d_ to approximate the system CF.[Fn fn4] Moreover, in the point charge electrostatic model (PCEM) approximation,^75^ we assume a priori the signs of the crystal field parameters, depending on the values observed for the typical angles (θ_*c*_,ϕ) of the SAP ligand field.

Assuming for the Zeeman term of eq 1 the isotropic Landé factor of the Dy^III^ free ion *g*_*j*_ = 4*/*3, and neglecting the intermolecular dipolar coupling, the CF parameters defined above are the only fitting parameters of the model. In Figure 3a,c, we observe that the experimental magnetization and susceptibility data are well reproduced by the model Hamiltonian. The low-temperature magnetization shows a sharp increase for a small static field and a close to linear increment above 1 T. Not reaching magnetization saturation even at high field is symptomatic of the presence of low-lying excited states, contributed through Van Vleck mechanisms. The low temperature χ*T* instead shows a sharp decrease, characteristic of the depopulation of the Zeeman-split crystal field levels, and a close-to-flat response above 50 K, reaching at room temperature a value compatible with that expected for a noninteracting Dy^III^ ion in the ^6^ *H*_15*/*2_, *S* = 5*/*2, *L* = 5, *J* = 15*/*2, and *g* = 4*/*3 configuration (14.17 cm^3^ K/mol).

According to the spin Hamiltonian that reproduces the data, the system eigenstates (Table S6) are structured with a |13/2⟩ ground-state doublet, unmixed with other CF states, because of the absence of off-diagonal matrix elements in the CF energy matrix (generated by *q* ≠ 0 parameters). The gap with the first excited doublet |11/2⟩ is 18.3 cm^–1^. This arrangement of states is in agreement with other examples of Dy complexes in *D*_4d_ symmetry with moderate CF,^76,77^ while in contrast to the configuration of strong axial CF, such as the one found (in a markedly different Dy^III^ coordination geometry) for the dysprosocenium complex, where the large *B*_0_^2^ CF term stabilizes the |15*/*2⟩ ground state.^8,9^ Additionally, we found for **Dy** ^**III**^ **Al** ^**III**^_**4**_ **[12-MC-4]** a modest overall CF splitting of the |*Jm*_*J*_⟩ multiplet (∼64 cm^–1^), compared to compounds with a more pronounced axial CF.

When the benzoate anions of **Dy** ^**III**^ **Al** ^**III**^_**4**_ **[12-MC-4]** are substituted with isophthalate anions, a dimeric 12-MC-4 complex is generated, **Dy** ^**III**^_**2**_ **Al** ^**III**^_**8**_ **[12-MC-4]**_**2**_, and the Dy^III^ ions of the dimer maintain an SAP coordination geometry (*D*_4d_). Therefore, for the fitting of the magnetometry data, we reasonably expect similar CF parameters obtained from the fitting of the **Dy** ^**III**^ **Al** ^**III**^_**4**_ **[12-MC-4]** data, which were thus chosen as a starting point for the fit of the dimer. Furthermore, the contribution arising from the dipolar coupling constant was calculated a priori from the distance between the Dy center of 7.12 Å, determined by X-ray diffraction. Once more, a satisfactory concordance is achieved between the experimental data for **Dy** ^**III**^_**2**_ **Al** ^**III**^_**8**_ **[12-MC-4]**_**2**_ and the theoretical curves derived from the model spin Hamiltonian, with similar crystal field parameters (Table S5), yielding an eigenstate spectrum comparable to those of the monomer. Indeed, the profile of *M*(*H*) and χ*T* is very close to what is measured for its monomer, with the exception of a factor of 2 in the magnetization values across the whole field range and a saturation value for the χ*T* product at room temperature, which matches the value expected for 2 isolated Dy^III^ ions (Figure 3b,d). Therefore, we conclude that because of the significant space separation between the Dy^III^ ions, the dipolar contribution here plays a minor contribution. To validate the modification in the modeled CF, we simulated the system eigenstates. The total CF multiplet splitting of **Dy** ^**III**^_**2**_ **Al** ^**III**^_**8**_ **[12-MC-4]**_**2**_ is slightly more than doubled with respect to **Dy** ^**III**^ **Al** ^**III**^_**4**_ **[12-MC-4]**, while the eigenstate spectra are mainly unchanged, with a ground state of the |13/2,13/2⟩ quartet, in which the four states |±13*/*2,∓13*/*2⟩ and |±13/2,±13/2⟩ are quasi-degenerate due to the small dipolar interaction. The gap with the first excited |13/2,11/2⟩, |11/2,13/2⟩ octet is about 14.1 cm^–1^. The eigenstates of the system as a function of the applied field are reported in the Supporting Information for both **Dy** ^**III**^ **Al** ^**III**^_**4**_ **[12-MC-4]** and **Dy** ^**III**^_**2**_ **Al** ^**III**^_**8**_ **[12-MC-4]**_**2**_ (Figure S3 and Table S6).

The small differences between the low-energy spectra of **Dy** ^**III**^ **Al** ^**III**^_**4**_ **[12-MC-4]** and **Dy** ^**III**^_**2**_ **Al** ^**III**^_**8**_ **[12-MC-4]**_**2**_ deduced from DC magnetometry reflect on the analogies observed in AC susceptibility measurements. No out-of-phase susceptibility χ″ is detected for either **Dy** ^**III**^ **Al** ^**III**^_**4**_ **[12-MC-4]** or **Dy** ^**III**^_**2**_ **Al** ^**III**^_**8**_ **[12-MC-4]**_**2**_ at temperatures above 2 K (Figure 4a,c); thus, they do not display SMM behavior in the experimentally accessible regime. However, a minor χ″ component is recovered with the application of a small static field (800 Oe). Similar behavior was noted for two Yb^III^Zn^II^_4_ [12-MC-4] complexes^78^ and a Dy^III^Cd^II^_16_ [12-MC-4]_2_[24-MC-4] system, where the Dy^III^ ion is sandwiched between two 12-MC-4 units.^79^ In these other systems, the Yb^III^ and Dy^III^ ions also have a square antiprism geometry (*D*_4d_) with little axial CF contribution as in the Dy-Al 12-MC-4 complexes. For the Dy^III^ and Yb^III^ 12-MC-4 systems, the presence of a χ″ component with the application of a small state field could be associated with the suppression of fast relaxation dynamics induced by the quantum tunneling of magnetization (QTM). However, even in an applied static field, neither complex shows any χ″ peak maxima above 2 K. This indicates that the relaxation rate exceeds the maximum detectable limit (1 kHz) within this temperature range (Figure 4b,d). In this context, the dimer **Dy** ^**III**^_**2**_ **Al** ^**III**^_**8**_ **[12-MC-4]**_**2**_ demonstrates a slightly more stable ground state, as indicated by its out-of-phase susceptibility at 2 K, which nearly reaches its maximum at the highest AC frequency measured. Conversely, the monomer **Dy** ^**III**^ **Al** ^**III**^_**4**_ **[12-MC-4]** does not exhibit a comparable approach to saturation within the same frequency range, implying faster relaxation processes, unresolved in the accessible frequency range.

For **Dy** ^**III**^ **Al** ^**III**^_**6**_ **[3.3.1] MCr,** the nine-coordinate Dy^III^ ion has a distorted spherical capped square antiprism geometry (*C*_4v_). The degree of deviation from the ideal geometry results in a continuous shape measure approaching 1.^56^ Here, the crystal field Hamiltonian is modeled with five parameters:

$$H_{\mathrm{CF}}=B_{0}^{2}{\hat{O}}_{0}^{2}+B_{0}^{4}{\hat{O}}_{0}^{4}+B_{4}^{4}{\hat{O}}_{4}^{4}+B_{0}^{6}{\hat{O}}_{0}^{6}+B_{4}^{6}{\hat{O}}_{4}^{6}$$

­(Table S5). However, because of the significant distortions of the *C*_4v_ symmetry group and the already large number of CF parameters, the fitting of the magnetometry data could suffer from overparameterization and the resulting correlations in CF parameters.[Fn fn5] Therefore, because of the stronger CF axiality (compared to monomer **Dy** ^**III**^ **Al** ^**III**^_**4**_ **[12-MC-4]**) promoted by the monocapping of the tetragonal symmetry, for the interpretation of the experimental measurements, we opted for an effective purely axial CF. This effective CF is sufficient to model adequately the magnetometry data (Figure 3e,g). Introducing off-axial components does not significantly improve the model fitting and affects its unambiguity. Moreover, due to the significantly distorted coordination, we did not constrain the sign of the CF parameters, depending on the expectation from the PCEM approximation for the exact *C*_4v_ group, which are strongly influenced by the characteristic angle of the ligand coordination.^75^ Again, for this monomer system, we neglected the intermolecular dipolar coupling. Therefore, the magnetometry data are fitted (Figure 3e,g) only with the effective axial CF defined above. In contrast to the results from **Dy** ^**III**^ **Al** ^**III**^_**4**_ **[12-MC-4]** and **Dy** ^**III**^_**2**_ **Al** ^**III**^_**8**_ **[12-MC-4]**_**2**_, the *M*(*H*) of this metallacryptate system shows a less sloped profile above 1 T, almost approaching saturation (Figure 3e). This is associated with a mitigated contribution of the low-lying excited states through Van Vleck mechanisms. A similar trend is instead found for the χ*T* product, where at low temperature a sharp decrease indicates the depopulation of the Zeeman split crystal field levels, and a plateau is observed above 50 K to the expected room temperature value for the noninteracting Dy^III^ ion in the ^6^ *H*_15*/*2_, *S* = 5*/*2, *L* = 5, *J* = 15*/*2 and *g* = 4*/*3 configuration (Figure 3g). The computed CF from fitting the magnetometry data reveals a significantly more pronounced *B*_0_^2^ term than what is observed in **Dy** ^**III**^ **Al** ^**III**^_**4**_ **[12-MC-4]** and **Dy** ^**III**^_**2**_ **Al** ^**III**^_**8**_ **[12-MC-4]**_**2**_, contributing to the stabilization of a |15*/*2⟩ ground state, characteristic of 4*f* systems under strong axial CF.^8,9,80–82^ Nonetheless, the CF in **Dy** ^**III**^ **Al** ^**III**^_**6**_ **[3.3.1] MCr** is insufficient for significantly isolating the ground state doublet from the excited states. Indeed, the first excited state is the |13*/*2⟩ multiplet, which is separated by a modest gap of ≈8 cm^–1^, while the total extent of the CF multiplet splitting is of ≈ 150 cm^–1^ (Figure S3 and Table S6).

For **Dy** ^**III**^_**2**_ **Al** ^**III**^_**6**_ **[18-MC-6]**, a [Dy^III^_2_(μ_3_-OH)_2_]^4+^ core is captured in the MC ring where both Dy^III^ ions are nine-coordinate. Compared to **Dy** ^**III**^ **Al** ^**III**^_**6**_ **[3.3.1] MCr**, the symmetry of the Dy^III^ ions in **Dy** ^**III**^_**2**_ **Al** ^**III**^_**6**_ **[18-MC-6]** is lowered to *C*_s_ as the geometry is best described as muffin. Unfortunately, the number of CF parameters in Steven’s notation derived from this very low symmetry coordination is incompatible with a reliable fitting. Thus, the most convenient approach for interpreting the magnetometry data of **Dy** ^**III**^_**2**_ **Al** ^**III**^_**6**_ **[18-MC-6]** is to define an effective CF model, starting from the three axial CF parameters obtained from **Dy** ^**III**^ **Al** ^**III**^_**6**_ **[3.3.1] MCr** and refining the fit without the introduction of additional off-axial parameters. From the comparison (Figure 3f,h), we conclude that this effective axial CF is sufficient to appropriately model the magnetometry data of **Dy** ^**III**^_**2**_ **Al** ^**III**^_**6**_ **[18-MC-6]**. Similarly to **Dy** ^**III**^_**2**_ **Al** ^**III**^_**8**_ **[12-MC-4]**_**2**_, because of the space separation between Dy^III^-ions in **Dy** ^**III**^_**2**_ **Al** ^**III**^_**6**_ **[18-MC-6]** (6.29 Å), the contribution to the spin Hamiltonian 1 of the Dy^III^–Dy^III^ interaction is assumed to be purely dipolar and the coefficient is calculated from eq 2. The best-fit curves reproduce both *M*(*H*) and χ*T*(*T*) (Figure 3f,h). The resulting effective CF maintains more robust axial components, with a dominant *B*_0_^2^ term, as also observed in **Dy** ^**III**^ **Al** ^**III**^_**6**_ **[3.3.1] MCr**, compared to what is found for the 12-MC-4 complexes. This contributes to the stabilization in the eas*y*-axis direction of a ground state |15*/*2,15*/*2⟩ quartet, in which the |±15/2,∓15/2⟩ and |±15/2,±15*/*2⟩ states are quasi-degenerate because of the small dipolar interaction. Similarly to **Dy** ^**III**^ **Al** ^**III**^_**6**_ **[3.3.1]**, a modest gap of 8.5 cm^–1^ is found with the first excited octet (|15/2,13/2⟩,|13/2,15/2⟩). Additionally, we found an enhanced CF multiplet total splitting of ≈510 cm^–1^ (Figure S3 and Table S6). The calculated system eigenstates as a function of the applied field are reported in the Supporting Information for both **Dy** ^**III**^ **Al** ^**III**^_**6**_ **[3.3.1]** and **Dy** ^**III**^_**2**_ **Al** ^**III**^_**6**_ **[18-MC-6]** (Table S6).

AC susceptibility measurements reveal that both **Dy** ^**III**^ **Al** ^**III**^_**6**_ **[3.3.1]** and **Dy** ^**III**^_**2**_ **Al** ^**III**^_**6**_ **[18-MC-6]** exhibit SMM behavior in zero applied field. However, at 2 K, the particularly fast relaxation rates for both compounds exceed the upper detection limit (1 kHz), as evidenced by the absence of a maximum in the measured out-of-phase susceptibility peak within the accessible frequency range. Also, for these complexes, the dinuclear compound exhibits a slower magnetization relaxation time in a zero static field, with a χ″ that approaches its maximum close to 1 kHz for **Dy** ^**III**^_**2**_ **Al** ^**III**^_**6**_ **[18-MC-6]** at 2 K. Upon application of a small static field (800 Oe), the relaxation rates of both systems fall within the accessible frequency window for most of the temperatures investigated (below ∼8 K). Under these conditions, the χ″ peak becomes observable and shifts to higher frequencies as the temperature increases. This trend indicates that thermally activated relaxation mechanisms involving coupling to molecular vibrations play a dominant role in accelerating the magnetization dynamics at higher temperatures. Notably, the slowest relaxation rate is observed for **Dy** ^**III**^ **Al** ^**III**^_**6**_ **[3.3.1]** at 2 K.

The main structural difference between the compounds, besides the class of MC, is the coordination geometry of the Dy^III^ ions. For **Dy** ^**III**^ **Al** ^**III**^_**4**_ **[12-MC-4]** and **Dy** ^**III**^_**2**_ **Al** ^**III**^_**8**_ **[12-MC-4]**_**2**_, the eight-coordinate Dy^III^ ions reside in a square antiprism geometry (*D*_4d_) with little axial contribution. However, for **Dy** ^**III**^ **Al** ^**III**^_**6**_ **[3.3.1] MCr** and **Dy** ^**III**^_**2**_ **Al** ^**III**^_**6**_ **[18-MC-6]**, the nine-coordinate Dy^III^ ions have a spherical capped square antiprism (*C*_4v_) and muffin (*C*_s_) geometry, respectively, with a more pronounced axial contribution to the crystal field. As extensively reported in the literature, a strong axial ligand field is a key ingredient enhancing the magnetic anisotropy, stabilizing the magnetic ground state, and effectively suppressing QTM. Consequently, it contributes to the SMM behavior of oblate ions such as Dy^III^.^23,81,83^ In fact, the strong axial ligand field has been identified as a crucial factor for the exceptionally high blocking temperatures observed in dysprosocenium complexes, which possess some of the largest *U*_eff_ values reported to date.^8,9,84^ Additionally, these systems benefit from a reduced coupling between the spin and molecular vibrations, further limiting relaxation dynamics.^23,81^ For **Dy** ^**III**^ **Al** ^**III**^_**4**_ **[12-MC-4]** and **Dy** ^**III**^_**2**_ **Al** ^**III**^_**8**_ **[12-MC-4]**_**2**_, the lack of a significant axial ligand field results in the nonexistence of SMM behavior in the absence of a static magnetic field, and a frequency-dependent out-of-phase magnetic susceptibility signal is only observed when a 800 Oe static magnetic field is applied.

For the investigated MCs, the axial ligands of **Dy** ^**III**^ **Al** ^**III**^_**6**_ **[3.3.1] MCr** and **Dy** ^**III**^_**2**_ **Al** ^**III**^_**6**_ **[18-MC-6]** allow the observation of SMM behavior under an applied static magnetic field of 800 Oe and the determination of the *U*_eff_ and the pre-exponential frequency factor (τ_0_) values. The AC data were analyzed with a Cole–Cole plot (Figure 5a,b) and fitted to the generalized Debye model to extract the relaxation times τ at different temperatures and their distribution α.^85,86^ Here, the parameters of the model were refined by fitting simultaneously both the generalized definitions for χ′(ω) and χ″(ω):

$$\chi^{\prime }(\omega )=\chi_{S}+\frac{\left(\chi_{T}-\chi_{S})(1+(\omega \tau {)}^{1-\alpha }\sin(\pi \alpha /2)\right)}{1+2(\omega \tau {)}^{1-\alpha }\sin(\pi \alpha /2)+(\omega \tau {)}^{2-2\alpha }}$$

$$\chi^{\prime \prime }(\omega )=\frac{\left(\chi_{T}-\chi_{S})(\omega \tau {)}^{1-\alpha }\cos(\pi \alpha /2\right)}{1+2(\omega \tau {)}^{1-\alpha }\sin(\pi \alpha /2)+(\omega \tau {)}^{2-2\alpha }}$$ 3

where α > 0 indicates a wide distribution of relaxation times τ and χ_S_, χ_T_ represents the adiabatic and isothermal limits of the susceptibility, respectively. Equation 3 correctly reproduced the experimental AC data. The resulting values for α vary in the range 0.36–0.42 and 0.52–0.68 for **Dy** ^**III**^ **Al** ^**III**^_**6**_ **[3.3.1] MCr** and **Dy** ^**III**^_**2**_ **Al** ^**III**^_**6**_ **[18-MC-6]**, respectively, indicating both a uniformly distributed relaxation process and a broader distribution of rates in the Dy^**III**^ dinuclear system (fitting parameters in Table S7). This broadening can be attributed to a more pronounced structural disorder in the dinuclear complex, which is reflected in the magnetic environment responsible for the relaxation dynamics of the systems.

**Figure 5:**
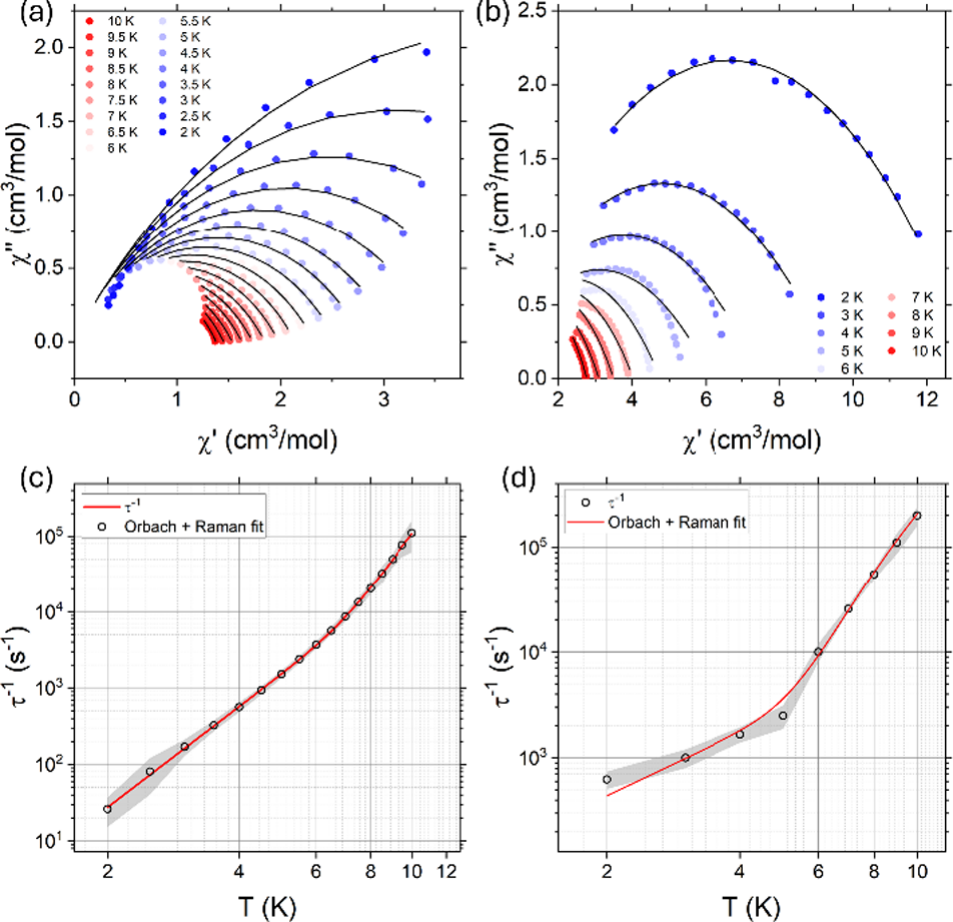
Temperature dependence of the Cole–Cole plots for (a) **Dy** ^**III**^ **Al** ^**III**^_**6**_ **[3.3.1] MCr** and (b) **Dy** ^**III**^_**2**_ **Al** ^**III**^_**6**_ **[18-MC-6]** in an applied static field of 800 Oe (dots). The solid line represents the fit with a generalized Debye model, as specified in the text. Relaxation rates (dots) extracted from the fitting as a function of temperature for (c) **Dy** ^**III**^ **Al** ^**III**^_**6**_ **[3.3.1] MCr** and (d) **Dy** ^**III**^_**2**_ **Al** ^**III**^_**6**_ **[18-MC-6],** respectively, are modeled (solid lines) with a combination of Raman and Orbach relaxation mechanisms, as detailed in the main text.

The relaxation rates were extracted by fitting the susceptibility as a function of the temperature (Figure 5c,d). These are expected to be governed by a single rate Arrhenius-like behavior τ_Orbach_^–1^ = τ_0_^–1^ *e* ^–*U*_eff_/*K*_B_ *T*^ in the higher temperature regime, where thermally activated mechanisms play a key role. Conversely, in the intermediate temperature regime, the relaxation rate is dominated by nonresonant Raman processes, which can be modeled with an effective power-law temperature dependence τ_Raman_^–1^ = *CT* ^*n*^.^87–89^ The relaxation rate is then fitted, in the corresponding temperature range, as the sum of these relaxation models. We extracted an effective energy barrier *U*_eff_ of 59 ± 2 cm^–1^ with τ_0_ = 2.5 ± 0.7 × 10^–8^ s and a Raman exponent *n* = 4.38 ± 0.04 with *C*_Raman_ = 1.3 ± 0.1 K^–*n*^ s^–1^ for complex **Dy** ^**III**^ **Al** ^**III**^_**6**_ **[3.3.1] MCr**, and *U*_eff_ = 38 ± 4 cm^–1^ with τ_0_ = 2.4 ± 1.1 × 10^–8^ s, and *n* = 2 ± 0.4 with *C*_Raman_ = 110 ± 60 K^–*n*^ s^–1^ for **Dy** ^**III**^_**2**_ **Al** ^**III**^_**6**_ **[18-MC-6]**.

In the Orbach regime, the differences between the complexes are less pronounced. The effective energy barrier is nearly halved in the dinuclear complex **Dy** ^**III**^_**2**_ **Al** ^**III**^_**6**_ **[18-MC-6]**, where this relaxation pathway becomes active at lower temperatures (see Figure 5c,d). For the mononuclear complex **Dy** ^**III**^ **Al** ^**III**^_**6**_ **[3.3.1] MCr**, the extracted barrier aligns well with the eigenstate spectrum obtained from fitting the DC susceptibility data. In contrast, for **Dy** ^**III**^_**2**_ **Al** ^**III**^_**6**_ **[18-MC-6]**, the observed discrepancy can be attributed to the invasive assumption of neglecting nonaxial contributions to the CF (Table S6). In the Raman regime, although it is characterized here by a limited number of data points, a tentative comparison can still be made. The markedly different Raman prefactor *C*_Raman_ between the two complexes may suggest a richer low-energy phonon spectrum for **Dy** ^**III**^_**2**_ **Al** ^**III**^_**6**_ **[18-MC-6]**, leading to a steeper phonon density of states (pDOS).^23,81^ Additionally, the significantly reduced Raman exponent in **Dy** ^**III**^_**2**_ **Al** ^**III**^_**6**_ **[18-MC-6]** (approaching the high-temperature limit^23,90,91^ where *n* ≈ 2[Fn fn6]) points to a higher-energy Debye cutoff, with low-lying optical modes remaining dispersive up to higher energies.^23,81^

#### 3.2.2 Comparison between Dy^III^–Al^III^ and Dy^III^–Ga^III^ MCs

While the magnetic properties of Ln^III^-Al^III^ MCs have not been reported before, the magnetic properties of several similar Dy^III^–Ga^III^ MCs have been previously investigated (Table 1). In 2019, Jiang, Shao, and co-workers reported a Dy^III^Ga^III^_4_ [12-MC-4] that displays slow magnetization relaxation under an applied DC magnetic field.^89^ However, unlike the slightly domed **Dy** ^**III**^ **Al** ^**III**^_**4**_ **[12-MC-4]**, the Dy^III^Ga^III^_4_ [12-MC-4] is significantly nonplanar as it is made with MC framework ligand 3-hydroxy-2-naphthanoic hydroxamic acid (H_3_napt). Four napt^3–^ comprise the MC unit, while two singly deprotonated H_2_napt^–^ ligands bridge between the ring Ga^III^ ions and the central Dy^III^ ion. The use of H_2_napt^–^ instead of benzoate likely leads to the nonplanarity of the 12-MC-4. In addition, the four-ring Ga^III^ ions do not lie in the same plane as in the **Dy** ^**III**^ **Al** ^**III**^_**4**_ **[12-MC-4]**. Furthermore, the central Dy^III^ of Dy^III^Ga^III^_4_ [12-MC-4] is nine-coordinate with a spherical tricapped trigonal prism geometry (*D*_3h_).

The central Dy^III^ ion binds to the oxime oxygen atoms of four napt^3–^, the oxime oxygen atoms of two H_2_napt^–^, the carbonyl oxygen atom of one H_2_napt^–^, and a bidentate nitrate ion. In **Dy** ^**III**^ **Al** ^**III**^_**4**_ **[12-MC-4]**, the central Dy^III^ ion is eight-coordinate with a square antiprism geometry (*D*_4d_). As in **Dy** ^**III**^ **Al** ^**III**^_**4**_ **[12-MC-4]**, the Dy^III^Ga^III^_4_ [12-MC-4] complexes do not exhibit a response in the out-of-phase magnetic susceptibility with zero applied magnetic field; however, a peak maximum was observed under applied DC magnetic fields between 200 and 2000 Oe. Under an applied DC magnetic field of 800 Oe, the authors determined the *U*_eff_ and τ_0_ values considering an Orbach-only process as 18.3 cm^–1^ and 2.2 × 10^–6^ s, respectively, and a process that considered all relaxation mechanisms (direct, QTM, Orbach, and Raman), as 18.18 cm^–1^ and 2.97 × 10^–6^ s, respectively. In 2019, Athanasopoulou, Rentschler, and co-workers reported a Dy^III^Ga^III^_8_ [12-MC-4]_2_ dimer where two Ga^III^_4_ 12-MC-4 units, based on shi^3–^ ligands, bind to one Dy^III^ ion.^68^ The Dy^III^ ion is captured in the central cavity of each MC to form a sandwich complex. The eight-coordinate Dy^III^ ion has a square antiprism geometry (*D*_4d_), similar to the **Dy** ^**III**^ **Al** ^**III**^_**4**_ **[12-MC-4]** and **Dy** ^**III**^_**2**_ **Al** ^**III**^_**8**_ **[12-MC-4]**_**2**_ complexes. For the Dy^III^Ga^III^_8_ [12-MC-4]_2_ dimer, the ground state is mainly composed by the |11/2> microstate with the first excited state being |13/2>, where instead for **Dy** ^**III**^ **Al** ^**III**^_**4**_ **[12-MC-4]** and **Dy** ^**III**^_**2**_ **Al** ^**III**^_**8**_ **[12-MC-4]**_**2**_ the ground states are |13/2> and |13/2,13/2⟩, respectively, and the first excited states are |11/2> and |13/2,11/2⟩, |11/2,13/2⟩, respectively. The Dy^III^Ga^III^_8_ [12-MC-4]_2_ dimer exhibited an out-of-phase magnetic susceptibility signal in zero applied DC magnetic field; however, peak maxima are not observed. Under a 1000 Oe applied DC magnetic field, peak maxima are observed, and considering both Orbach and Raman relaxation processes, the authors determined *U*_eff_ to be 27.1 cm^–1^ with a τ_0_ of 2.27 × 10^–8^ s. In 2018, Lutter, Pecoraro, and co-workers reported several Ln^III^Ga^III^_6_ [3.3.1] metallacryptates (Ln^III^ = Pr^III^, Nd^III^, and Sm^III^–Yb^III^) that are structurally similar to the **Dy** ^**III**^ **Al** ^**III**^_**6**_ **[3.3.1] MCr**.^67^ The Ln^III^Ga^III^_6_ [3.3.1] MCrs contains one H_2_shi^–^, one Hshi^2–^, and 7 shi^3–^ ligands and three triethylammonium countercations. The Ln^III^ ion is nine-coordinated with a spherical tricapped trigonal prism geometry (*D*_3h_). Conversely, the **Dy** ^**III**^ **Al** ^**III**^_**6**_ **[3.3.1] MCr** contains two H_2_shi^–^ and 7 shi^3–^ ligands and two pyridinium countercations, and the nine-coordinate Dy^III^ ion is in a spherical capped square antiprism geometry (*C*_4v_). However, the overall MCr connectivity is similar between both structures. The Nd^III^Ga^III^_6_, Dy^III^Ga^III^_6_, and Yb^III^Ga^III^_6_ [3.3.1] MCrs displayed a slow magnetization relaxation in the presence of a 1000 Oe (Nd^III^ and Yb^III^) or 750 Oe (Dy^III^) applied DC magnetic field; however, only the Dy^III^Ga^III^_6_ [3.3.1] MCr possessed a slow magnetization relaxation in zero applied field, though it is a weak response without a peak maximum in the out-of-phase magnetic susceptibility as in the **Dy** ^**III**^ **Al** ^**III**^_**6**_ **[3.3.1] MCr**. Using the 750 Oe applied DC field, the authors determined for the Dy^III^Ga^III^_6_ [3.3.1] MCr that the molecule contains one barrier to magnetization relaxation that follows an Orbach process. The *U*_eff_ value was 8.83 cm^–1^ with a τ_0_ of 3.6 × 10^–6^ s. In 2015, Pecoraro, Mallah, and co-workers reported a series of Ln^III^_2_Ga^III^_4_ MC-like complexes (Ln^III^ = Gd^III^, Tb^III^, Dy^III^, Er^III^, Y^III^, and Y^III^_0.9_Dy^III^_0.1_) built with H_3_shi ligands.^92^ The molecule is not an archetype MC as both N–O and oxygen-only connectivity exist between the metal ions, yet the complex can be considered a collapsed 16-MC-6 as it does not have a central cavity. Each Dy^III^ ion is eight-coordinated with a triangular dodecahedral geometry (*D*_2d_). Of the presented Dy^III^–Al^III^ MCs, the closest comparable structure would be **Dy** ^**III**^_**2**_ **Al** ^**III**^_**6**_ **[18-MC-6]**; however, the Dy^III^ ions in **Dy** ^**III**^_**2**_ **Al** ^**III**^_**6**_ **[18-MC-6]** are nine-coordinated with muffin geometry (*C*_s_). As in **Dy** ^**III**^_**2**_ **Al** ^**III**^_**6**_ **[18-MC-6]**, the Dy^III^_2_Ga^III^_4_ [16-MC-6] exhibited an out-of-phase magnetic susceptibility signal in zero applied DC magnetic field. However, the Dy^III^_2_Ga^III^_4_ [16-MC-6] complex possesses two relaxation processes: one process at lower temperatures (2–5 K) and one process at higher temperatures (10–14 K). At lower temperatures, the Dy^III^ ions are antiferromagnetically coupled, but an excited ferromagnetic state is accessible. The slow magnetization relaxation at lower temperatures is due to this populated excited state, and the authors determined *U*_eff_ to be 13 cm^–1^ with a τ_0_ of 3.6 × 10^–6^ s. At higher temperatures, the Dy^III^ ions are uncoupled and behave as isolated Dy^III^ ions with a *U*_eff_ of 18 cm^–1^ and a τ_0_ of 6.8 × 10^–6^ s. Though direct comparisons between the aluminum and gallium MC analogues are difficult, as the ligand sets, MC shape, Dy^III^ ground states, and experimental conditions are sometimes different, the Dy^III^–Al^III^ MC *U*_eff_ values tend to be larger than those of their Ga^III^ counterpart. For **Dy** ^**III**^ **Al** ^**III**^_**6**_ **[3.3.1] MCr** and **Dy** ^**III**^_**2**_ **Al** ^**III**^_**6**_ **[18-MC-6]**, the Dy^III^ ions have geometries with a greater axial component, which may be one of the contributing factors leading to the higher *U*_eff_ values.

**Table 1:** Comparison of SMM Parameters of Dy^III^–Al^III^ and Dy^III^–Ga^III^ Ms

**MC**	**Dy** ^**III**^ **ion coordination number and geometry**	***U***_**eff**_ **(cm** ^**–1**^ **)**	**τ**_**0**_ **(s)**
**Dy** ^**III**^ **Al** ^**III**^_**4**_ **[12-MC-4]**	8; square antiprism (*D*_4d_)	n.d.	n.d.
Dy^III^Ga^III^_4_ [12-MC-4][Bibr ref89]	9; tricapped trigonal prism geometry (*D*_3h_)	18.3	2.2 × 10^–6^
**Dy** ^**III**^_**2**_ **Al** ^**III**^_**8**_ **[12-MC-4]**_**2**_	8; square antiprism (*D*_4d_)	n.d.	n.d.
Dy^III^Ga^III^_8_ [12-MC-4]_2_ ^68^	8; square antiprism (*D*_4d_)	27.1	2.27 × 10^–8^
**Dy** ^**III**^ **Al** ^**III**^_**6**_ **[3.3.1] MCr**	9; capped square antiprism geometry (*C*_4v_)	59	2.5 × 10^–8^
Dy^III^Ga^III^_6_ [3.3.1] MCr^67^	9; tricapped trigonal prism geometry (*D*_3h_)	8.83	3.6 × 10^–6^
**Dy** ^**III**^_**2**_ **Al** ^**III**^_**6**_ **[18-MC-6]**	9; muffin (*C*_s_)	38	2.4 × 10^–8^
Dy^III^_2_Ga^III^_4_ [16-MC-6][Bibr ref92]	8; triangular dodecahedral geometry (*D*_2d_)	13 to 18	3.6 to 6.8 × 10^–6^

## 4 Conclusions

The four MC classes investigated reveal how systematic synthetic control through manipulation of stoichiometric ratios of the starting components can lead to the reliable formation of certain MCs, and within each structure, the coordination environment and geometries of the central Dy^III^ ions can be controlled by ligand scaffolding. For **Dy** ^**III**^ **Al** ^**III**^_**4**_ **[12-MC-4]** and **Dy** ^**III**^_**2**_ **Al** ^**III**^_**8**_ **[12-MC-4]**_**2**_, the Dy^III^ ions are sequestered in an eight-coordinate ligand environment with square antiprism geometry with little axial contribution to the ligand field. However, for **Dy** ^**III**^ **Al** ^**III**^_**6**_ **[3.3.1] MCr**, and **Dy** ^**III**^_**2**_ **Al** ^**III**^_**6**_ **[18-MC-6]**, the nine-coordinate Dy^III^ ions with spherical capped square antiprism or muffin geometry, respectively, have a greater axial component to the ligand field, and this axial contribution has a direct effect on the dynamic magnetic properties of the molecules.

From the perspective of SMM applications, all of the systems studied in this work reveal a slow relaxation of the magnetization at very low temperature and in an applied static field. However, only **Dy** ^**III**^ **Al** ^**III**^_**6**_ **[3.3.1]** and **Dy** ^**III**^_**2**_ **Al** ^**III**^_**6**_ **[18-MC-6]** display a slow relaxation of the magnetization in the absence of a static magnetic field. In addition, a peak in the out-of-phase susceptibility is observed in 800 Oe applied field with *U*_eff_ equal to 59 ± 2 and 38 ± 4 cm^–1^, and τ_0_ equal to 2.5 × 10^–8^ and 2.4 × 10^–8^ s for **Dy** ^**III**^ **Al** ^**III**^_**6**_ **[3.3.1]** and **Dy** ^**III**^_**2**_ **Al** ^**III**^_**6**_ **[18-MC-6]**, respectively. Thus, a key factor in determining the increased relaxation rate is attributed here to the axial nature of the crystal field on Dy^III^ ions. Therefore, to further improve the performance of these complexes, a valuable strategy would be to further engineer the Dy-ligand field by chemical substitution to deploy an axial structural modification of the molecule. Future AC susceptibility measurements at different applied fields and higher frequencies could also enable the disentangling of the contribution of different relaxation mechanisms. In addition, to gain greater insight into the magnetization relaxation mechanisms of the **Dy** ^**III**^_**2**_ **Al** ^**III**^_**6**_ **[18-MC-6]** system, diluted compounds where one of the Dy^III^ ions is replaced with a diamagnetic center, such as Y^III^ or Lu^III^, could also be performed. For example, diluted Dy/Y 18-MC-6 could better elucidate the relaxation processes occurring in the MC and allow for a better comparison of the molecules. Future work in our laboratories is directed toward the diluted versions of these MCs. Moreover, *ab initio* calculations of the complete crystal field tensors, phonon spectra, and vibrational density of states could serve as a valuable guide for rational structural design. Such a computational analysis would enable a more comprehensive understanding of the individual contributions of various relaxation mechanisms to the overall magnetization dynamics.

## Supplementary Material



## Footnotes

1 Bruker. Apex3 v2018.1-0, SAINT V8.38A; Bruker AXS Inc.: Madison, WI, 2018.

2 Sheldrick, G. M. SHELXL2018; University of Göttingen, Germany, 2018.

3 The main factors contributing to the experimental error are the sample mass (measured with a ±0.1 mg error) and the effective amount of solvent still incorporated in the crystal structure, which significantly influence the normalization into μ_B_ */*f.u*.* and cm^3^ K/mol units.

4 The implementation of a descent of symmetry approach to account for the small distortion found here would have caused an unnecessary overparametrization of the fitting Hamiltonian and to higher uncertainty on all of the obtained CF parameters.

5 The axial component fit is solid, while the accuracy in determining the nonaxial CF parameters is significantly reduced (surpassing the model sensitivity) and relies heavily on the initial fit parameters.

6 The lower bound for the fitted Raman exponent has been fixed to the high-temperature limit^90,91^ (high-energy Debye cutoff) of *n* = 2. It is worth noting that a rate approaching the linear dependence with the system temperature could also be associated with the establishment of direct relaxation processes (τ^–1^ = *AH* ^2^ *T*, with *H* static field and *A* prefactor), promoted by the applied static field and the small dipolar interaction, responsible for the admixture of excited states and the Kramers degeneracy breaking.^73^

## Notes

Crystallographic details, continuous shapes measures, and Dy and Al first-coordination sphere diagrams of Dy^III^_2_Al^III^_6_ [18-MC-6]; crystal field parameters, energy levels, eigenstates, and Cole–Cole parameters for investigated compounds (PDF)
