# Supplementary material for: Slow Relaxation of the Magnetization in Dysprosium–Aluminum Metallacrowns
Source: ACS Omega. 2025 Nov 14;10(46):56096–109. doi: 10.1021/acsomega.5c07724 (PMC12658786; doi:10.1021/acsomega.5c07724)
Supplement: Supplementary file 1 [file ao5c07724_si_001.pdf]

Supporting Information for
Slow relaxation of the magnetization in dysprosium-aluminum metallacrowns

Simone Chicco[1], Elena, Garlatti[1,2,3], Francesco Cugini[1], Rachel E. Rheam[4], Jordan R. Travis[4], Alyssa M. Hess[4], Matthias Zeller[5], Massimo Solzi,[1] Stefano Carretta[1,2,3*], Curtis M. Zaleski[4*]

1. Department of Mathematical, Physical and Computer Sciences, University of Parma, Parco Area delle Scienze 7/A, 43124 Parma, Italy
2. INFN-Sezione di Milano Bicocca, Gruppo Collegato di Parma, 43124 Parma, Italy
3. Consorzio Interuniversitario Nazionale per la Scienza e Tecnologia dei Materiali (INSTM), I-50121 Firenze, Italy
4. Department of Chemistry and Biochemistry, Shippensburg University, Shippensburg, PA 17257, USA
5. Department of Chemistry, Purdue University, West Lafayette, IN 47907, USA
(Dated: October 1, 2025)

Table S1. Crystallographic details for **$(Dy_2Al_6(H_2shi)_4(shi)_6(OH)_2(H_2O)_{2.24}(CH_3OH)_{1.759}(py)_2\cdot6.935py\cdot5.631CH_3OH\cdot1.2H_2O$, $Dy^{III}_2Al^{III}_6$ [18-MC-6]**.

Compound	**$Dy^{III}_2Al^{III}_6$ [18-MC-6]**
CCDC No.	2443596
Chemical formula	$C_{122.07}H_{131.12}Al_6Dy_2N_{18.93}O_{42.83}$
Formula weight (g/mol)	3035.58
Crystal system	Monoclinic
Temperature (K)	150(2)
λ (Å)	0.71073
Space group	$P2_1/n$
a (Å)	13.8025(7)
b (Å)	18.0241(8)
c (Å)	27.5528(13)
α (°)	90
β (°)	99.8896(19)
γ (°)	90
Volume (Å^3)	6752.7(6)
Z	2
Density (calculated), Mg/m^3	1.493
μ (mm^{-1})	1.227
F(000)	3097.3
Limiting indices	$-19 \le h \le 21$, $-27 \le k \le 27$, $-41 \le \ell \le 38$
Goodness-of-fit	1.022
R_1^a	0.0552 [$I>2\sigma(I)$]; 0.1062 (all data)
wR_2^b	0.1497 [$I>2\sigma(I)$]; 0.1807 (all data)

$^aR_1 = \Sigma(||F_o| - |F_c||)/\Sigma|Fo|$. $^bwR_2 = [\Sigma[w(F_o^2 - F_c^2)^2]/\Sigma[w(F^o)^2]]^{1/2}$; $w = 1/[\sigma^2(F_o^2) + (mp)^2 + np]$; $p = [\max(F_o^2,0) + 2F_c^2]/3$ (m and n are constants); $\sigma = [\Sigma[w(F_o^2 - F_c^2)^2]/(n - p)]^{1/2}$.

Table S2. Important metal-ligand bond lengths in **$Dy^{III}_2Al^{III}_6$ [18-MC-6]**.

	Bond Distance (Å)		Bond Distance (Å)
Dy1-O10	2.261(3)	Al2-O6	1.823(3)
Dy1-O13	2.310(3)	Al2-O12	1.828(3)
Dy1-O7	2.333(3)	Al2-O7	1.958(3)
Dy1-O8	2.350(3)	Al2-N2	1.981(4)
Dy1-O1	2.352(3)	Al2-N4	1.989(3)
Dy1-O17	2.372(4)	Al2-N6	2.138(10)
Dy1-O19	2.441(3)	Al3-O10	1.839(3)
Dy1-O16	2.581(3)	Al3-O13	1.844(3)
Dy1-O4	2.845(3)	Al3-O11	1.886(3)
Al1-O15	1.829(3)	Al3-O14	1.914(3)
Al1-O5	1.864(3)	Al3-O16	1.922(3)
Al1-O4	1.890(3)	Al3-O16	1.931(3)
Al1-O2	1.925(3)		
Al1-O1	1.970(3)		
Al1-N5	1.989(3)		

Table S3. Continuous Shapes Measures (CShM) values for the geometry about the nine-coordinate central Dy^{III} ion in **$Dy^{III}_2Al^{III}_6$ [18-MC-6]**

Shape	Dy1
Enneagon (D_{9h})	35.137
Octagonal Pyramid (C_{8v})	22.194
Heptagonal Bipyramid (D_{7h})	19.111
Johnson Triangular Cupola (J3; C_{3v})	15.760
Capped Cube (J8; C_{4v})	9.998
Spherical-relaxed Capped Cube (C_{4v})	8.305
Capped Square Antiprism (J10; C_{4v})	2.770
Spherical Capped Square Antiprism (C_{4v})	1.405
Tricapped Trigonal Prism (J51; D_{3h})	2.193
Spherical Tricapped Trigonal Prism (D_{3h})	1.842
Tridiminished Icosahedron (J63 ; C_{3v})	12.958
Hula-hoop (C_{2v})	12.027
**Muffin (C_s)**	**1.385**

Table S4. Continuous Shapes Measures (CShM) values for the geometry about the six-coordinate Al^{III} ions in **$Dy^{III}_2Al^{III}_6$ [18-MC-6]**

**Shape**	Al1	Al2	Al3
Hexagon (D_{6h})	32.165	32.251	32.843
Pentagonal Pyramid (C_{5v})	22.484	27.230	21.708
**Octahedron (O_h)**	**1.546**	**0.444**	**2.022**
Trigonal Prism (D_{3h})	10.074	14.501	8.940
Johnson Pentagonal Pyramid (J2; C_{5v})	26.184	30.854	25.861

Table S5. Crystal field parameters in cm^{-1} extracted for all the samples from the fitting of the magnetometry data. The error is attributed from the extrapolated error in the fitting parameter.

	B_0^2	B_0^4	B_0^6
**$Dy^{III}Al^{III}_4$ [12-MC-4]**	0	-8.8 ± 0.9	20.0 ± 0.3
**$Dy^{III}_2Al^{III}_8$ [12-MC-4]$_2$**	0	-18 ± 1.2	20.1 ± 0.3
**$Dy^{III}Al^{III}_6$ [3.3.1] MCr**	151 ± 11	14 ± 3	20 ± 1
**$Dy^{III}_2Al^{III}_6$ [18-MC-6]**	227 ± 14	39 ± 3	39.5 ± 1.5

Table S6. Energy levels and eigenstates composition in zero applied field, computed from the spin Hamiltonian parameters extracted by fitting the magnetometry data.

	Energy (cm^{-1})	*Eigenstate*
**$Dy^{III}Al^{III}_4$ [12-MC-4]**	0	$\| \pm 13/2 \rangle$
	18.3(5)	$\| \pm 11/2 \rangle$
	20.8(5)	$\| \pm 1/2 \rangle$
	33.3(6)	$\| \pm 3/2 \rangle$
	47.1(7)	$\| \pm 9/2 \rangle$
	50.1(7)	$\| \pm 5/2 \rangle$
	58.3(7)	$\| \pm 7/2 \rangle$
	63.7(8)	$\| \pm 15/2 \rangle$

	Energy (cm^{-1})	*Eigenstate*
**$Dy^{III}_2Al^{III}_8$ [12-MC-4]$_2$**	0	$\| \pm 13/2, \mp 13/2 \rangle, \| \pm 13/2, \pm 13/2 \rangle$
	14.1(4)	$\| \pm 11/2, \mp 13/2 \rangle, \| \pm 13/2, \mp 11/2 \rangle, 50\% \| \pm 13/2, \pm 11/2 \rangle + 50\% \| \pm 11/2, \pm 13/2 \rangle$
	28.2(6)	$\| \pm 11/2, \mp 11/2 \rangle, \| \pm 11/2, \pm 11/2 \rangle$
	30.2(6)	$\| \pm 13/2, \mp 1/2 \rangle, \| \pm 13/2, \pm 1/2 \rangle, \| \pm 1/2, \mp 13/2 \rangle, \| \pm 1/2, \pm 13/2 \rangle,$
	40.7(7)	$\| \pm 3/2, \mp 13/2 \rangle, \| \pm 3/2, \pm 13/2 \rangle, \| \pm 13/2, \mp 3/2 \rangle, \| \pm 13/2, \pm 3/2 \rangle$
	…	…
	134(1)	$\| \pm 7/2, \mp 15/2 \rangle, \| \pm 15/2, \mp 7/2 \rangle, \| \pm 15/2, \pm 7/2 \rangle, \| \pm 7/2, \pm 15/2 \rangle$
	152(1)	$\| \pm 15/2, \mp 15/2 \rangle, \| \pm 15/2, \pm 15/2 \rangle$

	Energy (cm^{-1})	*Eigenstate*
**$Dy^{III}Al^{III}_6$ [3.3.1] MCr**	0	$\| \pm 15/2 \rangle$
	7.8(3)	$\| \pm 13/2 \rangle$
	71.0(8)	$\| \pm 11/2 \rangle$
	125(1)	$\| \pm 1/2 \rangle$
	126(1)	$\| \pm 9/2 \rangle$
	137(1)	$\| \pm 3/2 \rangle$
	151(1)	$\| \pm 5/2 \rangle$
	152(1)	$\| \pm 7/2 \rangle$

	Energy (cm^{-1})	*Eigenstate*
**$Dy^{III}{}_2Al^{III}{}_6$ [18-MC-6]**	0	$\mid \pm 15/2, \mp 15/2 \rangle, \mid \pm 15/2, \pm 15/2 \rangle$
	8.5(3)	$50\% \mid \pm 13/2, \pm 15/2 \rangle + 50\% \mid \pm 15/2, \pm 13/2 \rangle$,<br>$50\% \mid \pm 13/2, \mp 15/2 \rangle + 50\% \mid \pm 15/2, \mp 13/2 \rangle$
	17.0(4)	$\mid \pm 13/2, \mp 13/2 \rangle, \mid \pm 13/2, \pm 13/2 \rangle$
	123(1)	$50\% \mid \pm 11/2, \pm 15/2 \rangle + 50\% \mid \pm 15/2, \pm 11/2 \rangle$,<br>$50\% \mid \pm 11/2, \mp 15/2 \rangle + 50\% \mid \pm 15/2, \mp 11/2 \rangle$
	131(1)	$50\% \mid \pm 11/2, \pm 13/2 \rangle + 50\% \mid \pm 13/2, \pm 11/2 \rangle$,<br>$50\% \mid \pm 11/2, \mp 13/2 \rangle + 50\% \mid \pm 13/2, \mp 11/2 \rangle$
	…	…
	495(5)	$50\% \mid \pm 7/2, \pm 5/2 \rangle + 50\% \mid \pm 5/2, \pm 7/2 \rangle$,<br>$25\% \mid \pm 7/2, \mp 5/2 \rangle + 25\% \mid \mp 7/2, \pm 5/2 \rangle + 25\% \mid \pm 5/2, \mp 7/2 \rangle + 25\% \mid$<br>$\mp 5/2, \pm 7/2 \rangle$
	511(5)	$\mid \pm 7/2, \mp 7/2 \rangle, \mid \pm 7/2, \pm 7/2 \rangle$

Table S7. Cole-Cole plot fit parameters.

	T (K)	χ_S *(cm³/mol)*	χ_T *(cm³/mol)*	α	τ *(s)*
**$Dy^{III}Al^{III}{}_6$ [3.3.1] MCr**	2	0 ± 0.04	9.6 ± 1.6	0.43 ± 0.03	$3.8 \pm 1.5 \times 10^{-2}$
	2.5	0 ± 0.03	6.2 ± 0.3	0.40 ± 0.02	$1.24 \pm 0.15 \times 10^{-2}$
	3	0 ± 0.02	4.9 ± 0.1	0.39 ± 0.01	$5.9 \pm 0.4 \times 10^{-3}$
	3.5	0 ± 0.01	4.1 ± 0.1	0.39 ± 0.01	$3.09 \pm 0.13 \times 10^{-3}$
	4	0 ± 0.01	3.6 ± 0.1	0.40 ± 0.01	$1.77 \pm 0.06 \times 10^{-3}$
	4.5	0 ± 0.01	3.15 ± 0.04	0.40 ± 0.01	$1.07 \pm 0.03 \times 10^{-3}$
	5	0 ± 0.01	2.82 ± 0.03	0.40 ± 0.01	$6.6 \pm 0.2 \times 10^{-4}$
	5.5	0 ± 0.01	2.56 ± 0.02	0.40 ± 0.01	$4.20 \pm 0.12 \times 10^{-4}$
	6	0 ± 0.01	2.34 ± 0.02	0.39 ± 0.01	$2.72 \pm 0.08 \times 10^{-4}$
	6.5	0 ± 0.01	2.15 ± 0.01	0.39 ± 0.01	$1.77 \pm 0.05 \times 10^{-4}$
	7	0 ± 0.01	1.99 ± 0.01	0.39 ± 0.01	$1.16 \pm 0.04 \times 10^{-4}$
	7.5	0 ± 0.01	1.85 ± 0.01	0.39 ± 0.01	$7.5 \pm 0.3 \times 10^{-5}$
	8	0 ± 0.01	1.74 ± 0.01	0.38 ± 0.01	$4.9 \pm 0.2 \times 10^{-5}$
	8.5	0 ± 0.01	1.63 ± 0.01	0.38 ± 0.01	$3.1 \pm 0.2 \times 10^{-5}$
	9	0 ± 0.01	1.54 ± 0.01	0.38 ± 0.01	$2.0 \pm 0.1 \times 10^{-5}$
	9.5	0 ± 0.01	1.455 ± 0.004	0.37 ± 0.02	$1.3 \pm 0.1 \times 10^{-5}$
	10	0 ± 0.01	1.38 ± 0.003	0.35 ± 0.02	$0.9 \pm 0.1 \times 10^{-5}$

	T (K)	χ_S *(cm³/mol)*	χ_T *(cm³/mol)*	α	τ *(s)*
**$Dy^{III}{}_2Al^{III}{}_6$ [18-MC-6]**	2	0 ± 0.02	13.3 ± 0.1	0.60 ± 0.01	$1.6 \pm 0.2 \times 10^{-3}$
	3	0 ± 0.01	9.62 ± 0.05	0.65 ± 0.01	$1.0 \pm 0.1 \times 10^{-3}$
	4	0 ± 0.02	7.5 ± 0.1	0.67 ± 0.01	$0.6 \pm 0.1 \times 10^{-3}$
	5	0 ± 0.04	6.3 ± 0.2	0.68 ± 0.02	$0.4 \pm 0.1 \times 10^{-3}$
	6	0 ± 0.02	4.82 ± 0.06	0.63 ± 0.01	$0.10 \pm 0.03 \times 10^{-3}$
	7	0 ± 0.01	4.07 ± 0.03	0.61 ± 0.01	$0.39 \pm 0.03 \times 10^{-4}$
	8	0 ± 0.01	3.52 ± 0.02	0.58 ± 0.01	$1.8 \pm 0.2 \times 10^{-5}$
	9	0 ± 0.01	3.12 ± 0.01	0.57 ± 0.01	$0.9 \pm 0.1 \times 10^{-5}$
	10	0 ± 0.01	2.78 ± 0.01	0.52 ± 0.01	$0.5 \pm 0.1 \times 10^{-5}$

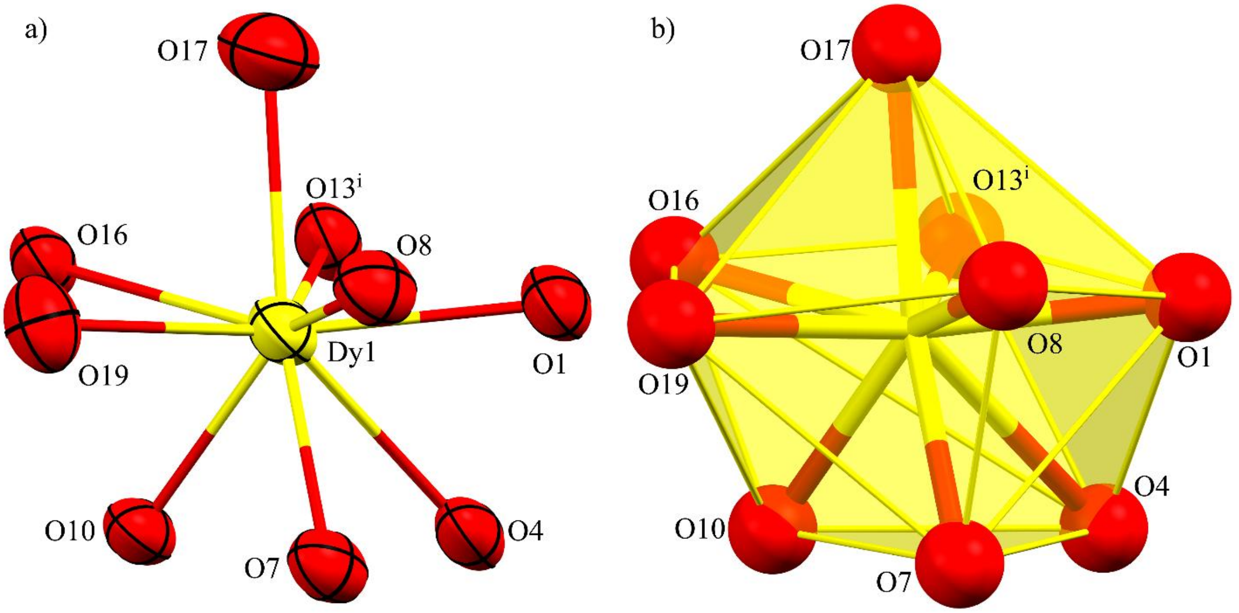

Figure S1. First coordination sphere of Dy1 with muffin geometry (C_s) from **$Dy^{III}{}_2Al^{III}{}_6$ [18-MC-6]**. (a) ellipsoid plot at the 50% level (b) polyhedral view. Color scheme: Dy^{III} – yellow and oxygen – red. [symmetry code: (i) -x+1, -y+1, -z]

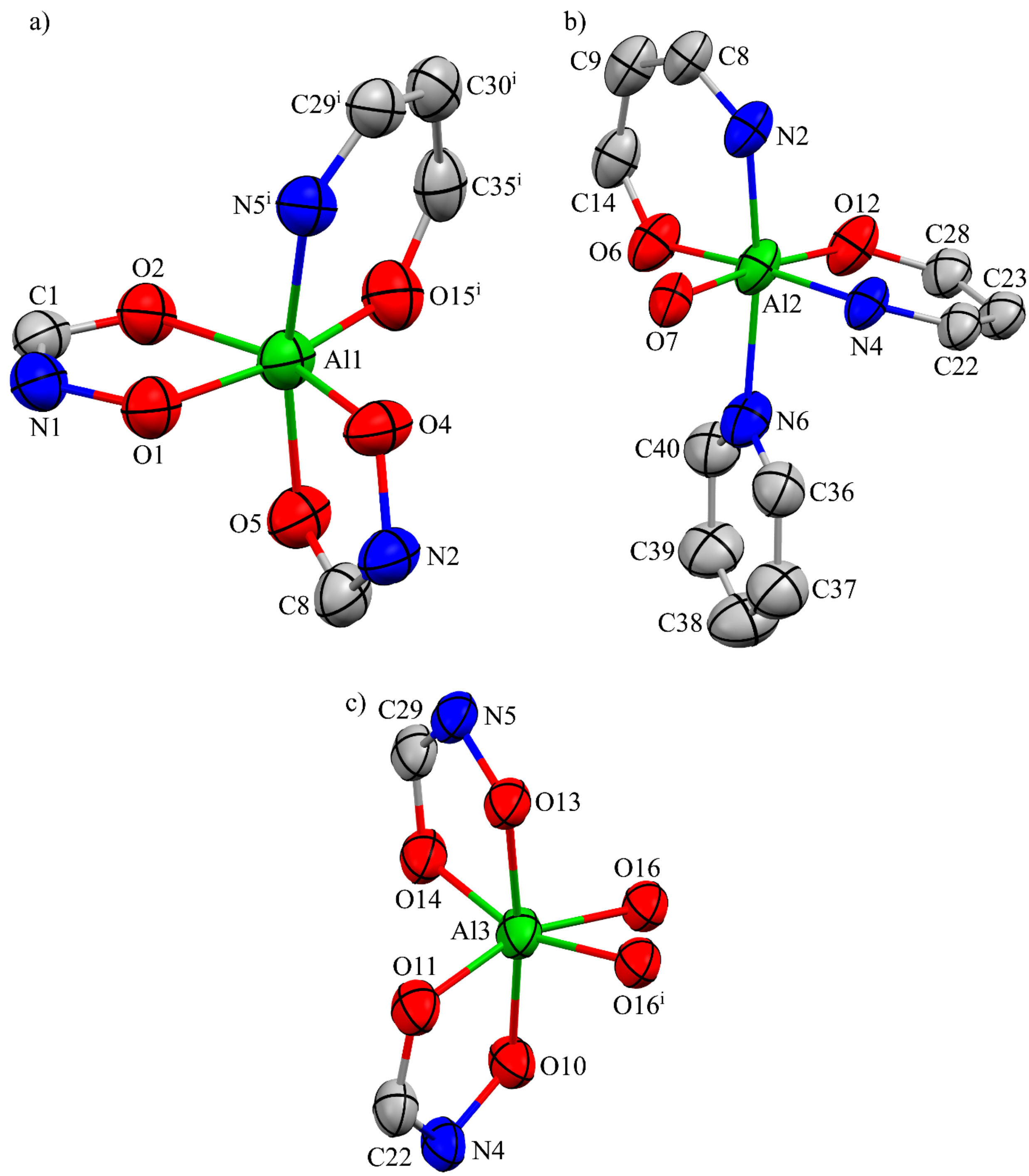

Figure S2. Coordination geometries for the Al^{III} ions of **$Dy^{III}_2Al^{III}_6$ [18-MC-6]**: (a) Al1 – octahedral, Δ; (b) Al2 – octahedral, Λ; (c) Al3 – octahedral, Δ. Color scheme: Al^{III} – green, oxygen – red, nitrogen – blue, and carbon - gray. [symmetry code: (i) -x+1, -y+1, -z]

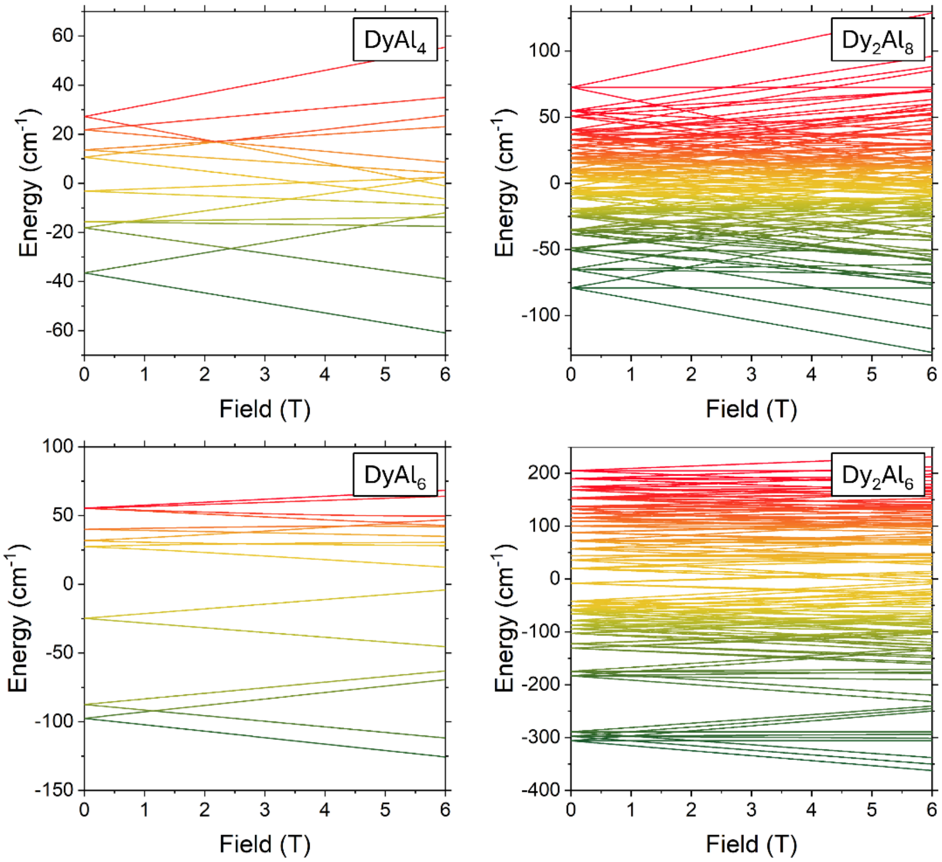

Figure S3. Eigenstates as a function of the applied static field, computed from the parameters of the spin Hamiltonian, refined by fitting the magnetometry data. Here, the Zeeman term of the spin Hamiltonian is responsible for the splitting of the states degeneracy. (top left: **$Dy^{III}Al^{III}_4$ [12-MC-4]**; top right: **$Dy^{III}_2Al^{III}_8$ [12-MC-4]$_2$**; bottom left: **$Dy^{III}Al^{III}_6$ [3.3.1] MCr**; bottom right: **$Dy^{III}_2Al^{III}_6$ [18-MC-6]**.)
